# Supplementary material for: Separation and Identification of Antioxidants and Aldose Reductase Inhibitors in Lepechinia meyenii (Walp.) Epling
Source: Plants (Basel). 2021 Dec 15;10(12):2773. doi: 10.3390/plants10122773 (PMC8707451; doi:10.3390/plants10122773)
Supplement: Supplementary file 1 [file plants-10-02773-s001.zip › plants-1502366-supplementary.pdf]

plants

MDPI

Supplementary Data

Separation and Identification of Antioxidants and Aldose Reductase Inhibitors in *Lepechinia meyenii* (Walp.) Epling

Guanglei Zuo [1], Kang-Hoon Je [2], Yanymee N. Guillen Quispe [3], Kyong-Oh Shin [1], Hyun Yong Kim [1], Kang Hyuk Kim [1], Paul H. Gonzales Arce [4], Soon Sung Lim [1,2,5,*]

[1] Department of Food Science and Nutrition, Hallym University,1 Hallymdeahak-gil, Chuncheon, 24252, Republic of Korea; guangleizuo@foxmail.com (G.Z.); tlsruddhek@hallym.ac.kr (K.O.S.); khy9514@nate.com (H.Y.K.); M20023@hallym.ac.kr (K.H.K.)

[2] Institute of Korean Nutrition, Hallym University, 1 Hallymdeahak-gil, Chuncheon 24252 Republic of Korea; jekh@hallym.ac.kr (K.H.J.)

[3] Department of Molecular Medicine and Biopharmaceutical Sciences, Graduate School of Convergence Science and Technology, Seoul National University, Seoul, 151742, Republic of Korea; yany24@snu.ac.kr (Y.N.G.Q.)

[4] Laboratorio de Florística, Departamento de Dicotiledóneas, Museo de Historia Natural – Universidad Nacional Mayor de San Marcos, Avenida Arenales 1256, Lima 14-0434, Peru; pgonzalesarce@hotmail.com (P.H.G.A.)

[5] Institute of Natural Medicine, Hallym University, 1 Hallymdeahak-gil, Chuncheon, 24252, Republic of Korea

* Correspondence: limss@hallym.ac.kr (S.S.L.); Tel.: +82-33-248-2133; Fax: +82-33-256-3420

Supplementary data list:

Table S1. ^{1}H-NMR data of rosmarinic acid derivatives from *L. meyenii* and chemical esterification (δ in ppm, *J* in Hz).

Table S2. NMR data of compounds **2**, **4**, and **6** isolated from *L. meyenii*.

Table S3. Calibration curve, detection/quantification limits, and precision of rosmarinic acid.

Table S4. The recovery rate of rosmarinic acid in spike recovery test

Figure S1. Chemical synthesis and HSCCC purification of ethyl rosmarinate.

Figure S2. Chemical synthesis and HSCCC purification of propyl rosmarinate.

Figure S3. ^{1}H-NMR (400 MHz, MeOD-d_4) spectroscopy of caffeic acid (**1**).

Figure S4.1. Part 1 of ^{1}H-NMR (600 MHz, DMSO-d_6) spectroscopy of hesperidin (**2**).

Figure S4.2. Part 2 of ^{1}H-NMR (600 MHz, DMSO-d_6) spectroscopy of hesperidin (**2**).

Figure S4.3. Part 1 of ^{1}H-^{1}H COSY NMR (600 MHz, DMSO-d_6) spectroscopy of hesperidin (**2**).

Figure S4.4. Part 2 of ^{1}H-^{1}H COSY NMR (600 MHz, DMSO-d_6) spectroscopy of hesperidin (**2**).

Figure S4.5. Part 3 of ^{1}H-^{1}H COSY NMR (600 MHz, DMSO-d_6) spectroscopy of hesperidin (**2**).

Figure S4.6. Part 4 of ^{1}H-^{1}H COSY NMR (600 MHz, DMSO-d_6) spectroscopy of hesperidin (**2**).

Figure S4.7. ESI-MS/MS (negative ion) spectroscopy of hesperidin (**2**).

Figure S4.8. ESI-MS/MS (positive ion) spectroscopy of hesperidin (**2**).

Figure S5.1. ^{1}H-NMR (600 MHz, MeOH-d_4) spectroscopy of rosmarinic acid (**3**).

Figure S5.2. ^{1}H-^{1}H COSY NMR (600 MHz, MeOH-d_4) spectroscopy of rosmarinic acid (**3**).

Figure S6.1. Part 1 of ^{1}H-NMR (600 MHz, DMSO-d_6) spectroscopy of diosmin (**4**).

Figure S6.2. Part 2 of ^{1}H-NMR (600 MHz, DMSO-d_6) spectroscopy of diosmin (**4**).

Figure S6.3. Part 1 of ^{1}H-^{1}H COSY NMR (600 MHz, DMSO-d_6) spectroscopy of diosmin (**4**).

Figure S6.4. Part 2 of ^{1}H-^{1}H COSY NMR (600 MHz, DMSO-d_6) spectroscopy of diosmin (**4**).

Figure S6.5. Part 3 of ^{1}H-^{1}H COSY NMR (600 MHz, DMSO-d_6) spectroscopy of diosmin (**4**).

Figure S6.6. ESI-MS/MS (negative ion) spectroscopy of diosmin (**4**).

Figure S6.7. ESI-MS/MS (positive ion) spectroscopy of diosmin (**4**).

Figure S7. ^{1}H-NMR (600 MHz, MeOH-d_4) spectroscopy of methyl rosmarinate (**5**).

Figure S8.1. ^{1}H-NMR (600 MHz, DMSO-d_6) spectroscopy of diosmetin (**6**).

Figure S8.2. ^{13}C-NMR (150 MHz, DMSO-d_6) spectroscopy of diosmetin (**6**).

Figure S8.3. ESI-MS/MS (negative ion) spectroscopy of diosmetin (**6**).

Figure S8.4. ESI-MS/MS (positive ion) spectroscopy of diosmetin (**6**).

Figure S9. ^{1}H-NMR (400 MHz, MeOH-d_4) spectroscopy of *n*-butyl rosmarinate (**7**).

Figure S10. ^{1}H-NMR (400 MHz, MeOH-d_4) spectroscopy of synthesized ethyl rosmarinate (**S1**).

Figure S11. ^{1}H-NMR (400 MHz, MeOH-d_4) spectroscopy of synthesized propyl rosmarinate (**S2**).

Table S1. ^{1}H-NMR data of rosmarinic acid derivatives from *L. meyenii* and chemical esterification (*δ* in ppm, *J* in Hz).

Position	Caffeic acid (1)[1,2]	Rosmarinic acid (3)	Methyl rosmarinate (5)	Ethyl rosmarinate (S1)	Propyl rosmarinate (S2)	*n*-Butyl rosmarinate (7)
1						
2	7.03 (d, 2.0)	7.04 (d, 2.0)	7.04 (d, 1.7)	7.04 (d, 2.0)	7.03 (d, 1.6)	7.04 (d, 2.0)
3						
4						
5	6.77 (d, 8.0)	6.77 (d, 8.2)	6.78 (d, 8.1)	6.77 (d, 8.0)	6.77 (d, 8.0)	6.77 (d, 7.6)
6	6.93 (dd, 8.0, 2.0)	6.94 (dd, 8.1, 2.1)	6.94 (dd, 8.0, 1.7)	6.95 (dd, 8.0, 2.0)	6.95 (dd, 8.0, 1.6)	6.95 (dd, 7.6, 1.6)
7	7.52 (d, 16.0)	7.54 (d, 15.9)	7.55 (d, 15.9)	7.55 (d, 16.0)	7.55 (d, 15.6)	7.56 (d, 16.0)
8	6.21 (d, 16.0)	6.26 (d, 15.9)	6.26 (d, 15.9)	6.28 (d, 16.0)	6.27 (d, 15.6)	6.27 (d, 16.0)
9						
1'	-					
2'	-	6.74 (d, 2.0)	6.71 (d, 1.6)	6.71 (d, 2.0)	6.71 (d, 2.0)	6.71 (d, 2.0)
3'	-					
4'	-					
5'	-	6.69 (d, 8.2)	6.69 (d, 8.2)	6.69 (d, 8.0)	6.69 (d, 8.0)	6.69 (d, 8.0)
6'	-	6.61 (dd, 8.2, 2.0)	6.57 (dd, 8.2, 1.6)	6.57 (dd, 8.0, 2.0)	6.57 (dd, 8.0, 2.0)	6.57 (dd, 8.0, 2.0)
7'a	-	3.09 (dd, 14.4, 4.3)	3.05 (dd, 14.2, 5.2)	3.02 (m)[3]	3.03 ("d"[4], 7.2)[3]	3.03 ("d", 6.8)[3]
7'b		3.00 (dd, 14.4, 8.4)	3.01 (dd, 14.2, 7.5)			
8'	-	5.18 (dd, 8.4, 4.3)	5.19 (dd, 7.5, 5.2)	5.14 ("t", 6.4)	5.15 ("t", 7.2)	5.15 ("t", 6.8)
9'	-					
1''	-	-	3.69 (s)	4.14 (q, 7.2)	4.04 (m)	4.07 (t, 6.4)
2''	-	-	-	1.20 (t, 7.2)	1.59 (m)	1.56 (m)
3''	-	-	-	-	0.88 (t, 7.2)	1.29 (m)
4''	-	-	-	-	-	0.88 (t, 7.2)

[1] Caffeic acid (**1**), rosmarinic acid (**3**), methyl rosmarinate (**5**) and *n*-butyl rosmarinate (**7**) were separated from *Lepechinia meyenii* (Walp.) Epling, while ethyl (**S1**) and propyl (**S2**) rosmarinates were synthesized.
[2] The ^{1}H NMR data of rosmarinic acid and methyl rosmarinate were recorded by 600 MHz NMR, while others were recorded by 400 MHz NMR. MeOD-d_4 was used as NMR solvent.
[3] Overlapped signals.
[4] Seems like doublet ("d") or triplet ("t").

Table S2. NMR data of compounds **2**, **4**, and **6** isolated from *L. meyenii*.

Peak number	^{1}H-NMR, δ (ppm)	^{13}C-NMR, δ (ppm)	Structural assignment
**2**	6.96-6.89 (3H, overlapped, 2'-, 5'-, 6'-H), 6.14 (1H, d, *J* = 2.10 Hz, 8-H), 6.12 (1H, d, *J* = 2.07 Hz, 6-H), 5.49 (1H, dd, *J* = 7.58, 2.98 Hz, 2-H), 4.98 (1H, d, *J* = 7.91 Hz, 1''-H), 4.52 (1H, s, 1'''-H), 3.79 (1H, d, *J* = 1.39 Hz, 6''a-H), 3.77 (3H, s, -OCH$_3$), 3.64 (1H, d, *J* = 11.38 Hz, 2'''-H), 3.54 (1H, dd, *J* = 8.00, 7.93 Hz, 3''-H), 3.43-3.38 (3H, overlapped, 3'''-, 5''-, 5'''-H), 3.28-3.26 (2H, overlapped, 6''b-, 3 (S)-H), 3.22 (1H, m, 2''-H, 2''-H), 3.17-3.13 (2H, overlapped, 4'''-, 4''-H), 2.76 (1H, dd, *J* = 9.41, 2.95 Hz, 3 (R)-H), 1.09 (3H, d, *J* = 6.12 Hz, 6'''-H)	-	Hesperidin
**4**	7.57 (1H, dd, *J* = 8.52 Hz, 2.27 Hz, 6'-H), 7.45 (1H, d, *J* = 2.29 Hz, 2'-H), 7.14 (1H, d, *J* = 8.69 Hz, 5'-H), 6.82 (1H, s, 3-H), 6.76 (1H, d, *J* = 2.07 Hz, 8-H), 6.48 (1H, d, *J* = 2.13 Hz, 6-H), 5.08 (1H, d, *J* = 7.48 Hz, 1''-H), 5.08 (1H, d, *J* = 7.48 Hz, 1''-H), 4.55 (1H, brs, 1'''-H), 3.87 (3H, s, -OCH$_3$), 3.85 (1H, d, *J* = 10.07 Hz, 6''a -H), 3.60 (1H, ddd, *J* = 6.97 Hz, 6.41 Hz, 1.23 Hz, 2'''-H), 3.48-3.39 (3H, m, 5''-, 3''-, 5'''-H), 3.32-3.25 (2H, m, 6''b-, 2''-H), 3.18-3.13 (3H, m, 3'''-, 4''-, 4'''-H), 1.08 (3H, d, *J* = 6.20 Hz, 6'''-H)	-	Diosmin
**6**	7.55 (1H, brd, *J* = 6.74 Hz, 6'-H), 7.43 (1H, brs, 2'-H), 7.09 (1H, brd, *J* = 7.51 Hz, 5'-H), 6.76 (1H, brs, 3-H), 6.47 (1H, brs, 8-H), 6.20 (1H, brs, 6-H), 3.87 (3H, s, -OCH$_3$).	182.17 (C-4), 164.69 (C-7), 164.00 (C-2), 161.93 (C-9), 157.79 (C-5), 151.62 (C-4'), 147.27 (C-3'), 151.62 (C-4'), 147.27 (C-3'), 123.46 (C-6'), 119.19 (C-1'), 113.41 (C-2'), 112.63 (C-5'), 104.21 (C-3), 103.99 (C-10), 99.35 (C-6), 94.39 (C-8), 56.24 (-OCH$_3$)	Diosmetin

Note: The ^{1}H NMR data of compounds **2**, **4**, and **6** were recorded by 600 MHz NMR and the ^{13}C NMR of compound **6** was recorded by 151 MHz NMR. DMSO-d_6 was used as the NMR solvent. The assignment labels of the severely overlapped sugar protons between 3.0 ppm and 3.5 ppm for compounds **2** and **4** were tentatively assigned according to their ^{1}H-^{1}H COSY NMR data.

Table S3. Calibration curve, detection/quantification limits, and precision of rosmarinic acid

**Parameter**		**Rosmarinic acid**
Calibration curve, r^2		y=14.013x+4.7301, r^2=1.000
Linear range (μg/mL)		0.39-400.00
Limit of detection (*S*/*N*=3)		0.15
Limit of quantification (*S*/*N*=10)		0.39
Intra-day variability (n=6), RSD (%)	12.50 μg/mL	3.53
	100.00 μg/mL	1.24
Inter-day variability (n=3), RSD (%)	12.50 μg/mL	4.85
	100.00 μg/mL	1.47

Note: RSD is the abbreviation of relative standard deviation.

Table S4. The recovery rate of rosmarinic acid in spike recovery test

C_0 (μg/mL)	V_0 (mL)	C_1 (μg/mL)	V_1 (mL)	C_2 (μg/mL)	V_2 (mL)	Recovery (%)
25	0.20	13.54±0.05	0.20	19.95±0.05	0.40	105.46±0.40
100	0.20	13.54±0.05	0.20	109.94±0.19	0.40	103.17±0.19

Note: values of C_1, C_2, and Recovery (%) were presented as mean ± standard deviation.

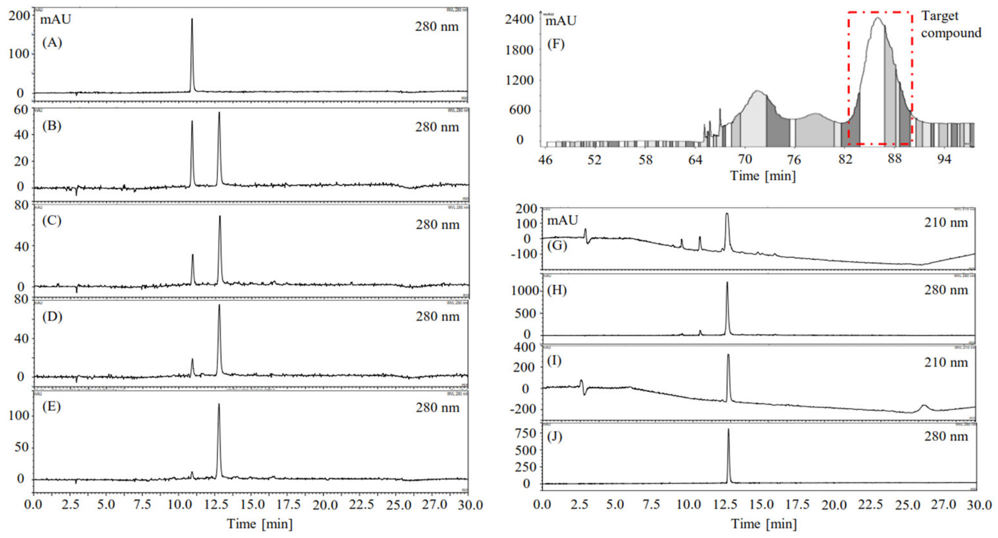

Figure S1. Chemical synthesis and HSCCC purification of ethyl rosmarinate. HPLC spectrum (280 nm) of the reaction mixture of rosmarinic acid and anhydrous ethanol at (A) 0th h, (B) 14th h, (C) 24th h, (D) 37th h, and (E) 72nd h reaction time. The reaction solution was filtrated by a 0.45 μm syringe filter when the reaction took 72 hrs and evaporated to dryness to get ethyl rosmarinate (G, 210 nm; H, 280 nm). The impure ethyl rosmarinate was then purified by HSCCC (F, 210 nm) to obtain high purity ethyl rosmarinate (I, 210 nm; J, 280 nm).

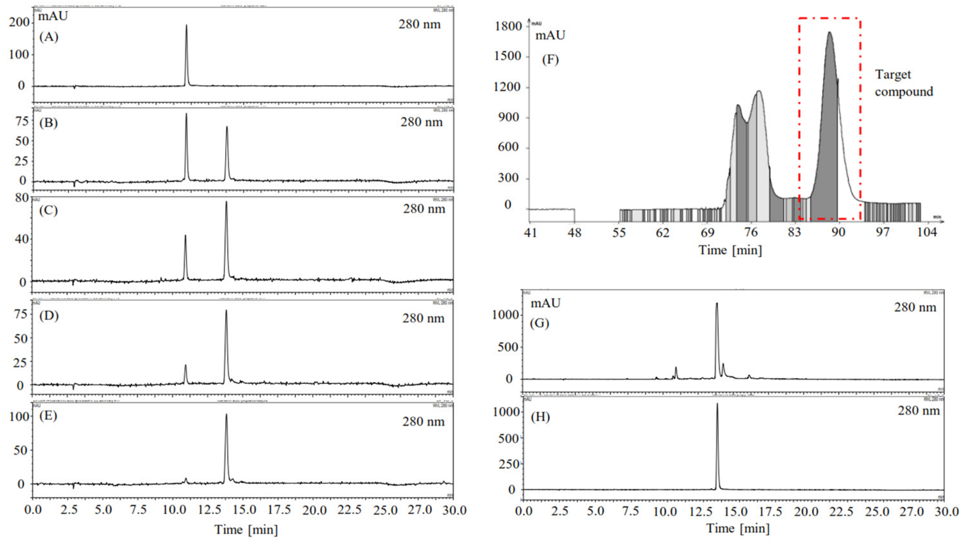

Figure S2. Chemical synthesis and HSCCC purification of propyl rosmarinate. HPLC spectrum (280 nm) of the reaction mixture of rosmarinic acid and anhydrous *n*-propanol at (A) 0th h, (B) 14th h, (C) 24th h, (D) 37th h, and (E) 72nd h reaction time. The reaction solution was filtrated by a 0.45 μm syringe filter when the reaction took 72 hrs and evaporated to dryness to get propyl rosmarinate (G, 280 nm). The impure propyl rosmarinate was then purified by HSCCC (F, 280 nm) to obtain high purity propyl rosmarinate (H, 280 nm).

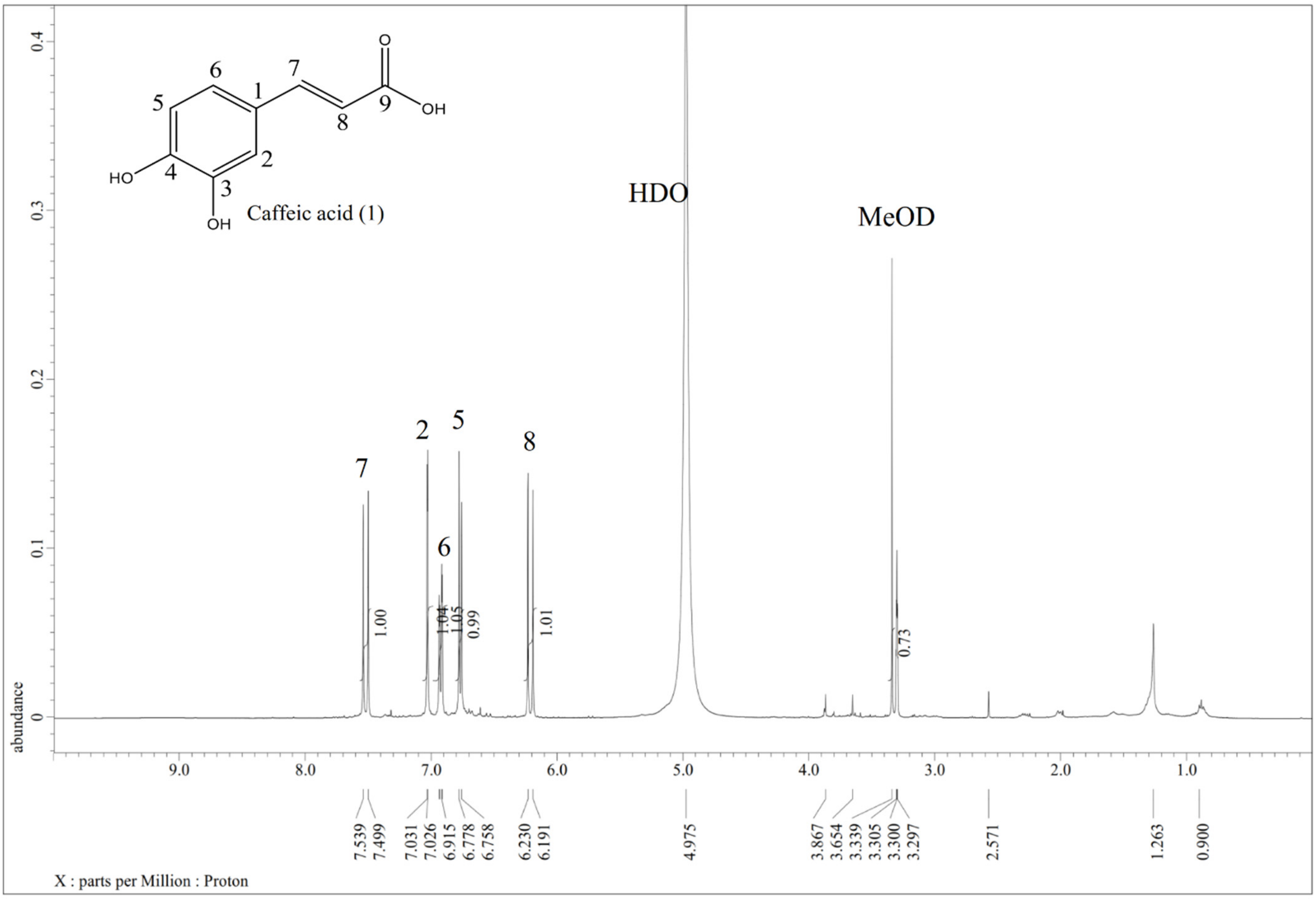

Figure S3. ^{1}H-NMR (400 MHz, MeOD-d_4) spectroscopy of caffeic acid (**1**).

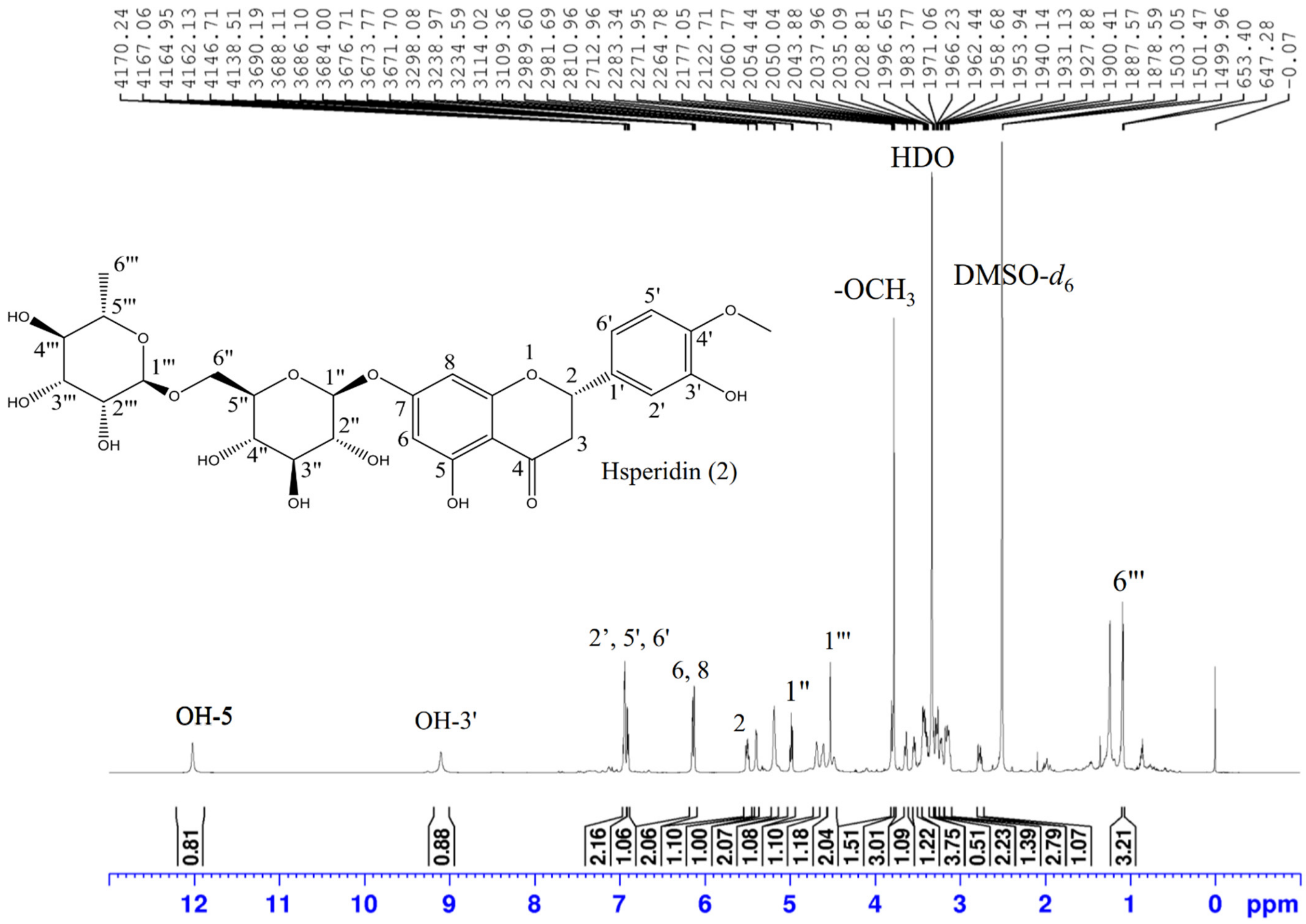

Figure S4.1. Part 1 of ^{1}H-NMR (600 MHz, DMSO-d_6) spectroscopy of hesperidin (**2**).

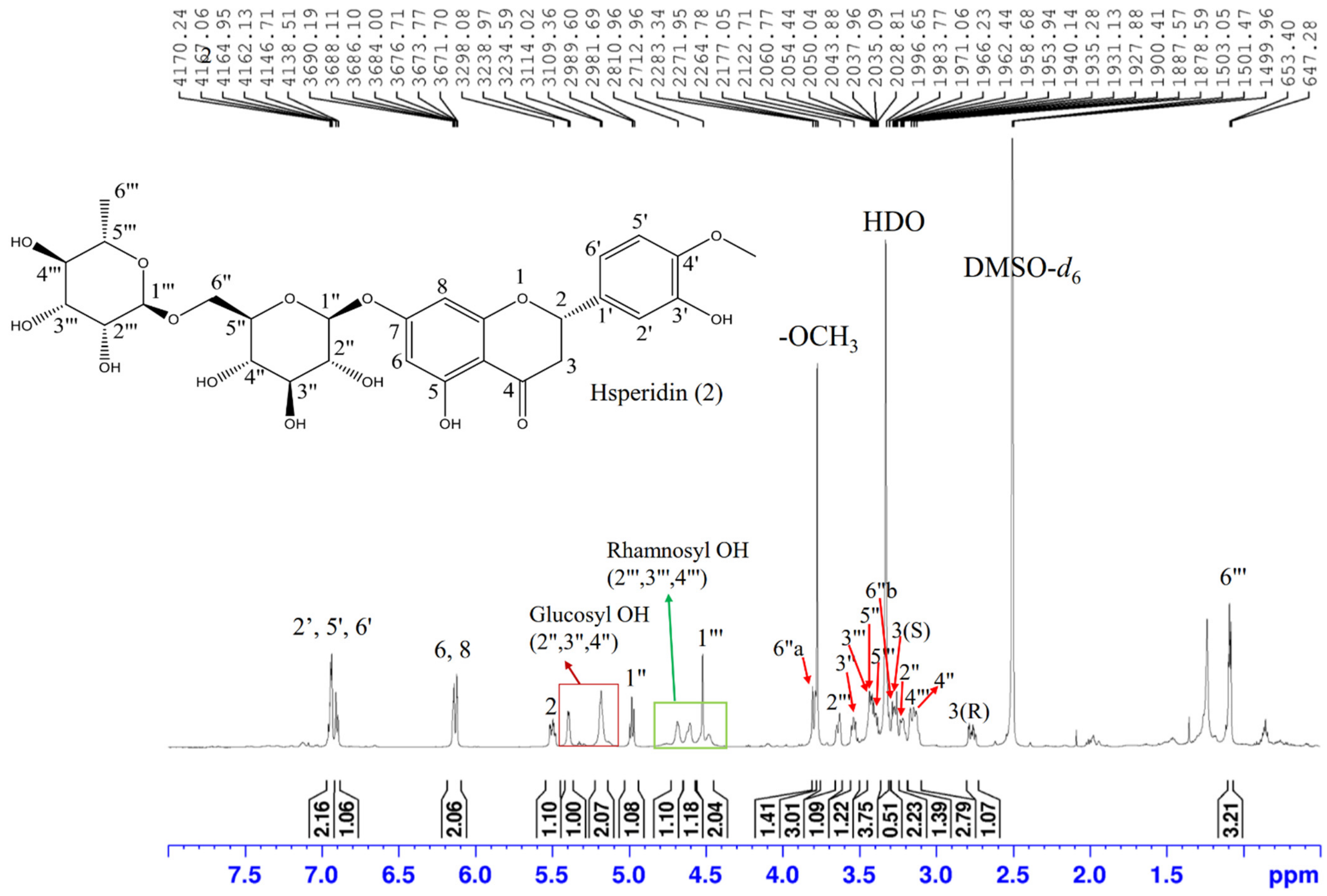

Figure S4.2. Part 2 of ^{1}H-NMR (600 MHz, DMSO-d_6) spectroscopy of hesperidin (**2**). Please note that the assignment labels of the severely overlapped sugar protons between 3.0 ppm and 3.5 ppm for compound **2** were tentatively assigned according to its ^{1}H-^{1}H COSY NMR data.

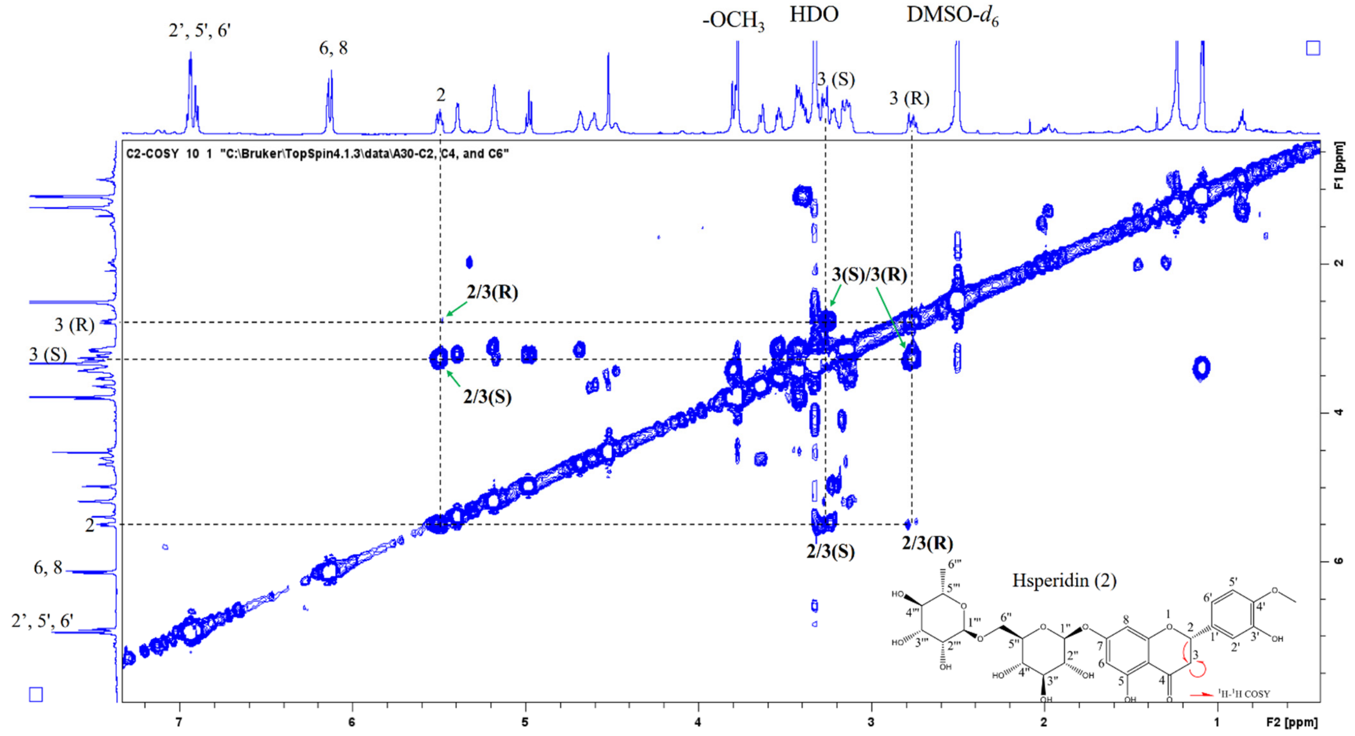

Figure S4.3. Part 1 of ^{1}H-^{1}H COSY NMR (600 MHz, DMSO-d_6) spectroscopy of hesperidin (**2**).

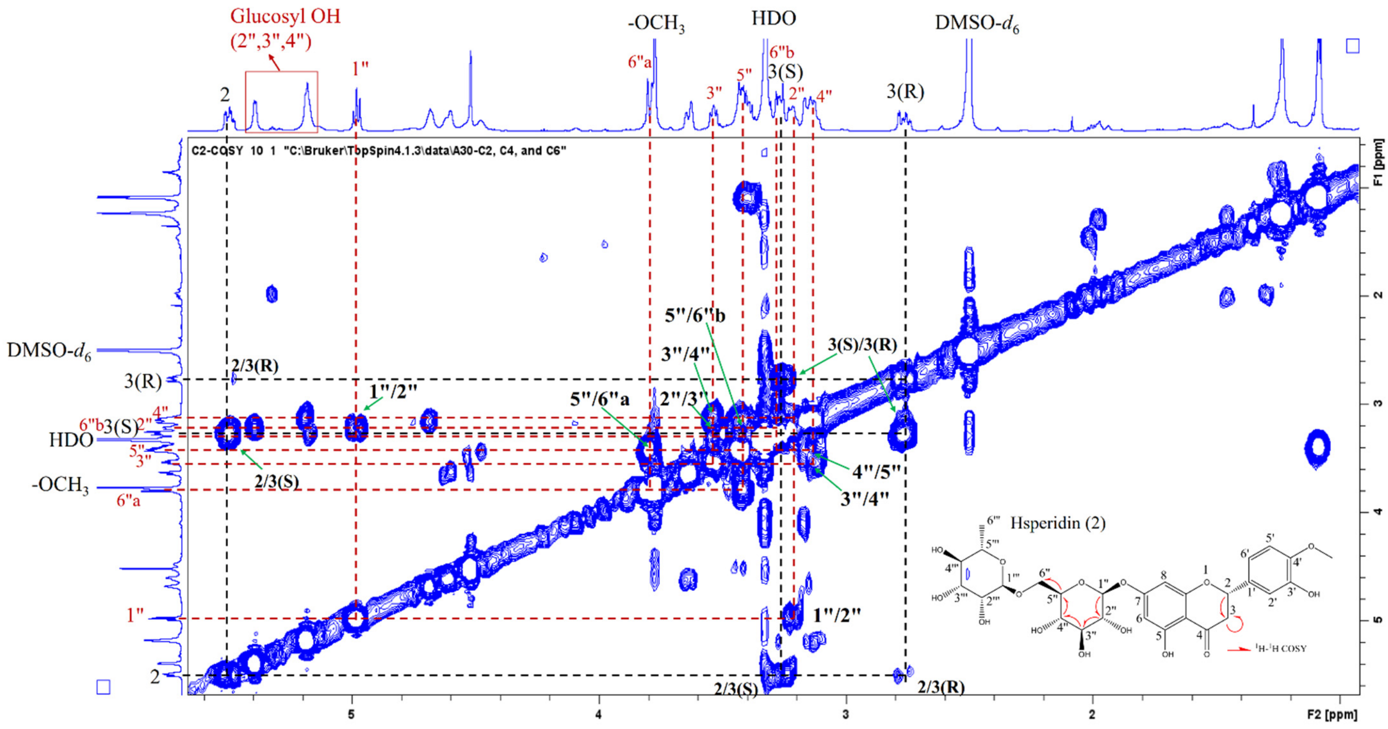

Figure S4.4. Part 2 of ^{1}H-^{1}H COSY NMR (600 MHz, DMSO-d_6) spectroscopy of hesperidin (**2**). Please note that the assignment labels of the severely overlapped sugar protons between 3.0 ppm and 3.5 ppm for compound **2** were tentatively assigned according to the crosspeaks in its ^{1}H-^{1}H COSY NMR data.

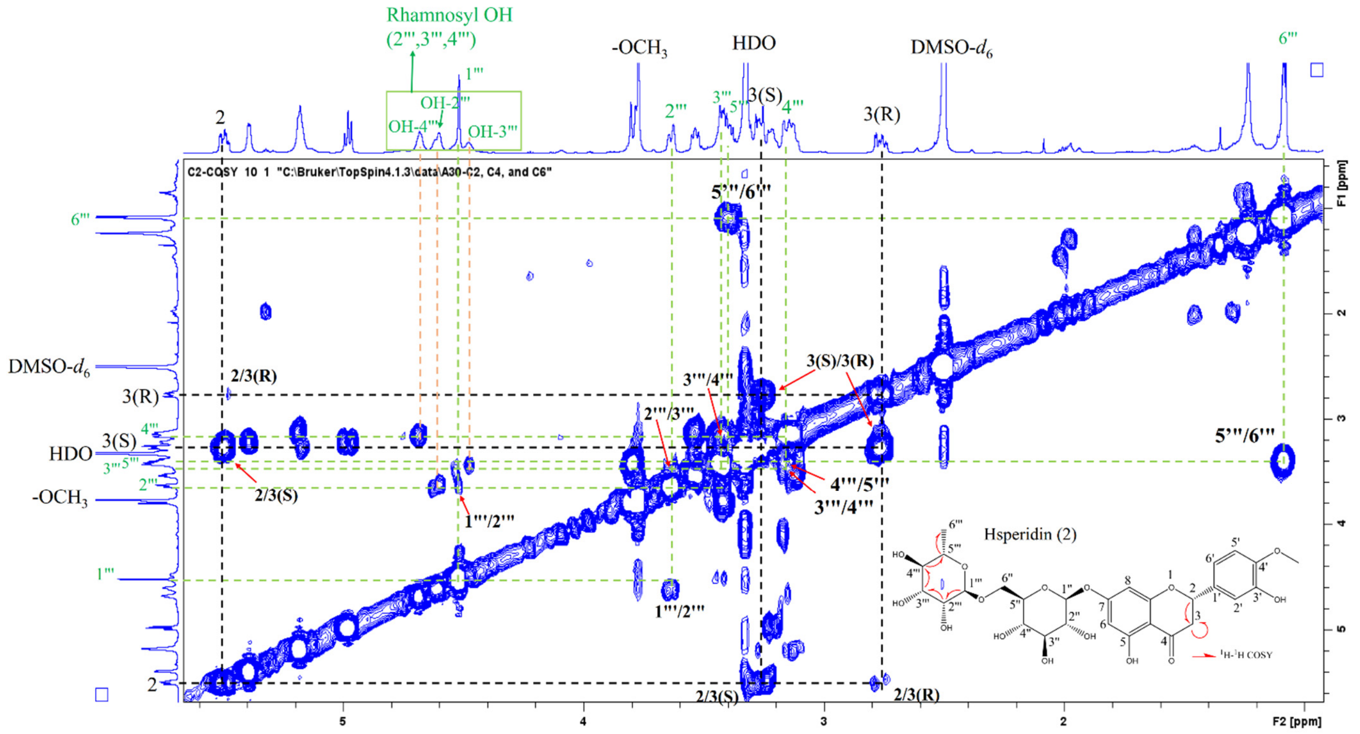

Figure S4.5. Part 3 of ¹H-¹H COSY NMR (600 MHz, DMSO-d_6) spectroscopy of hesperidin (**2**). Please note that the assignment labels of the severely overlapped sugar protons between 3.0 ppm and 3.5 ppm for compound **2** were tentatively assigned according to the crosspeaks in its ¹H-¹H COSY NMR data.

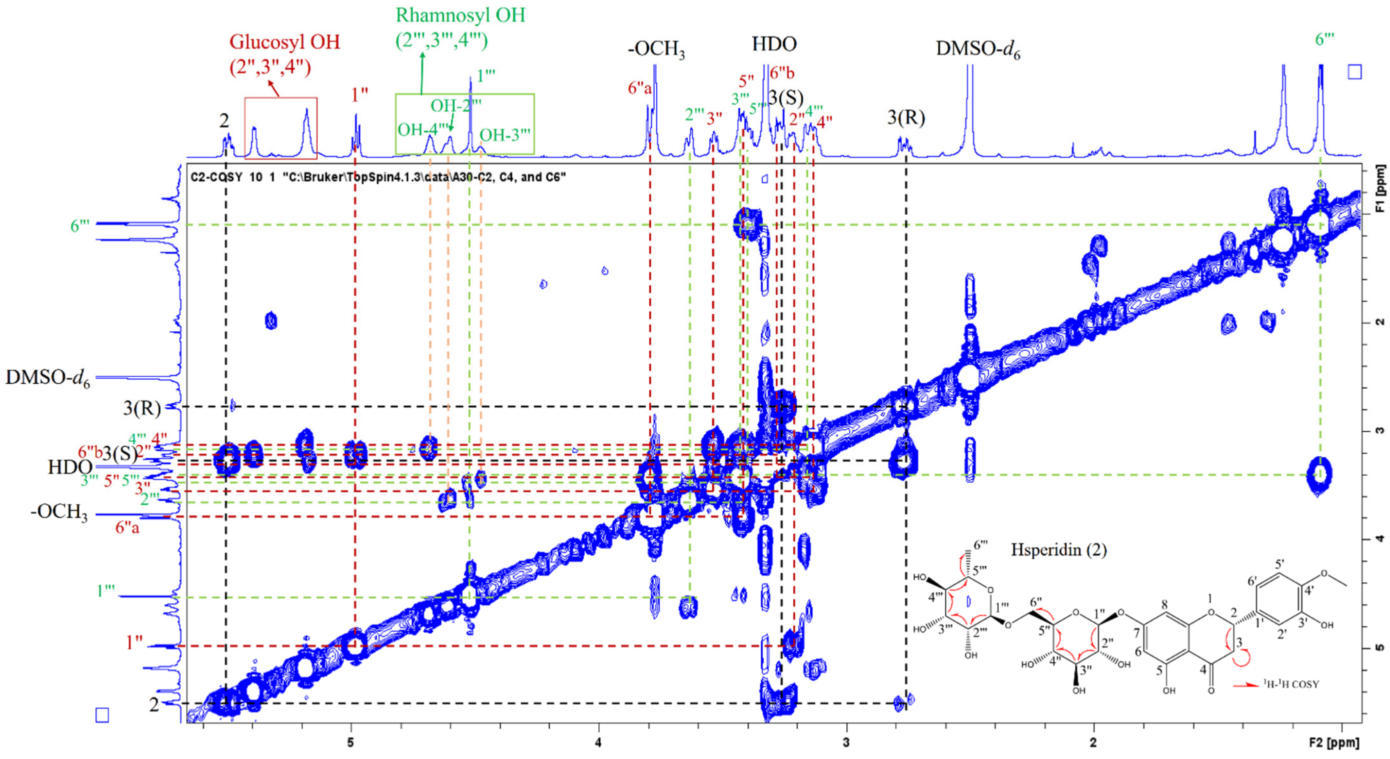

Figure S4.6. Part 4 of ¹H-¹H COSY NMR (600 MHz, DMSO-d_6) spectroscopy of hesperidin (**2**). Please note that the assignment labels of the severely overlapped sugar protons between 3.0 ppm and 3.5 ppm for compound **2** were tentatively assigned according to the crosspeaks in its ¹H-¹H COSY NMR data.

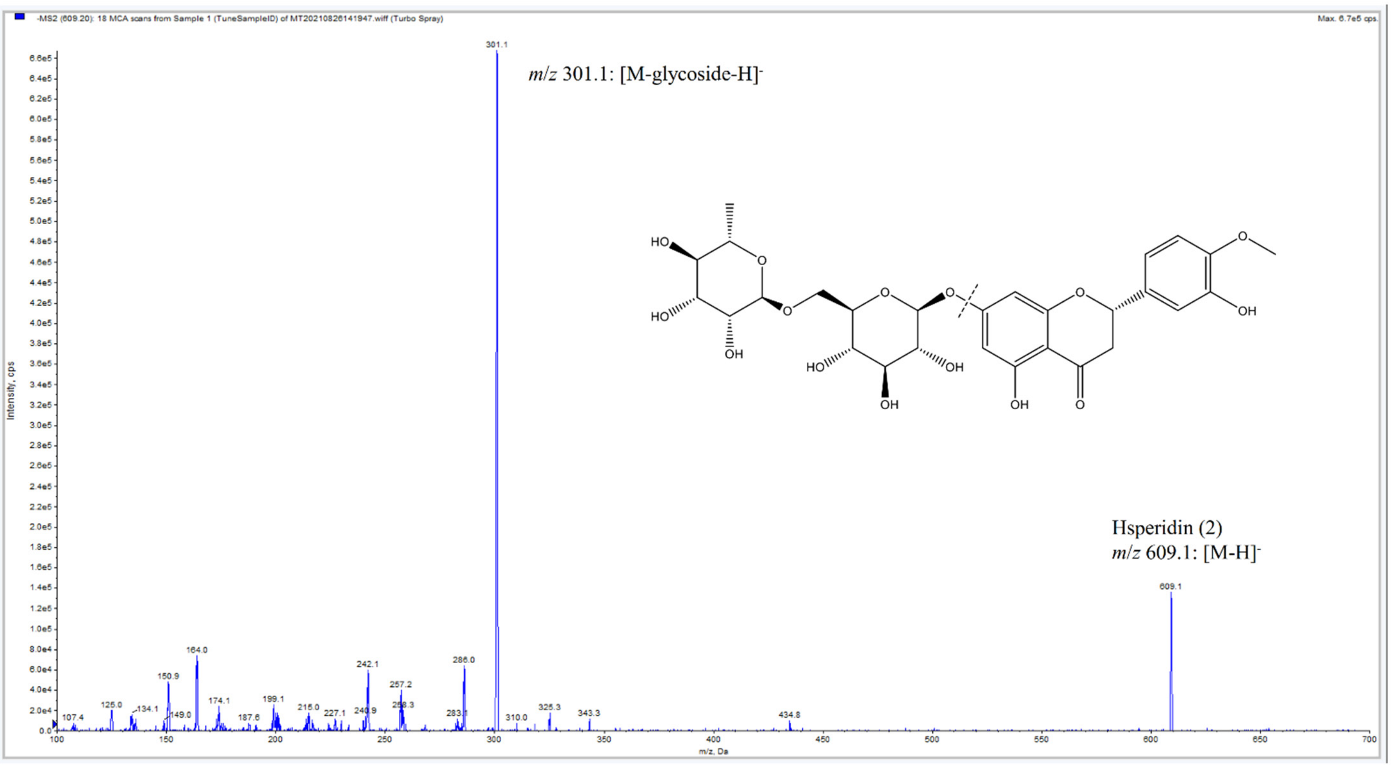

Figure S4.7. ESI-MS/MS (negative ion) spectroscopy of hesperidin (**2**).

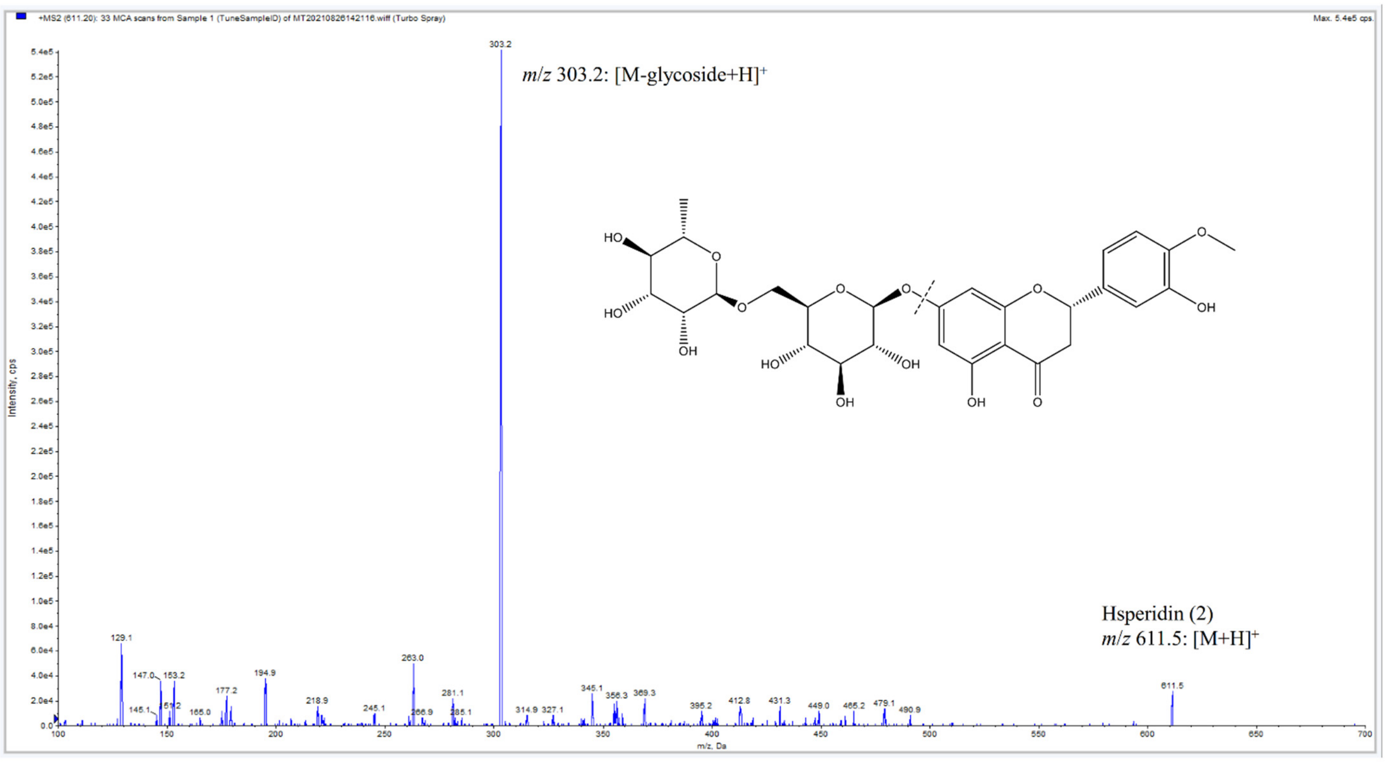

Figure S4.8. ESI-MS/MS (positive ion) spectroscopy of hesperidin (**2**).

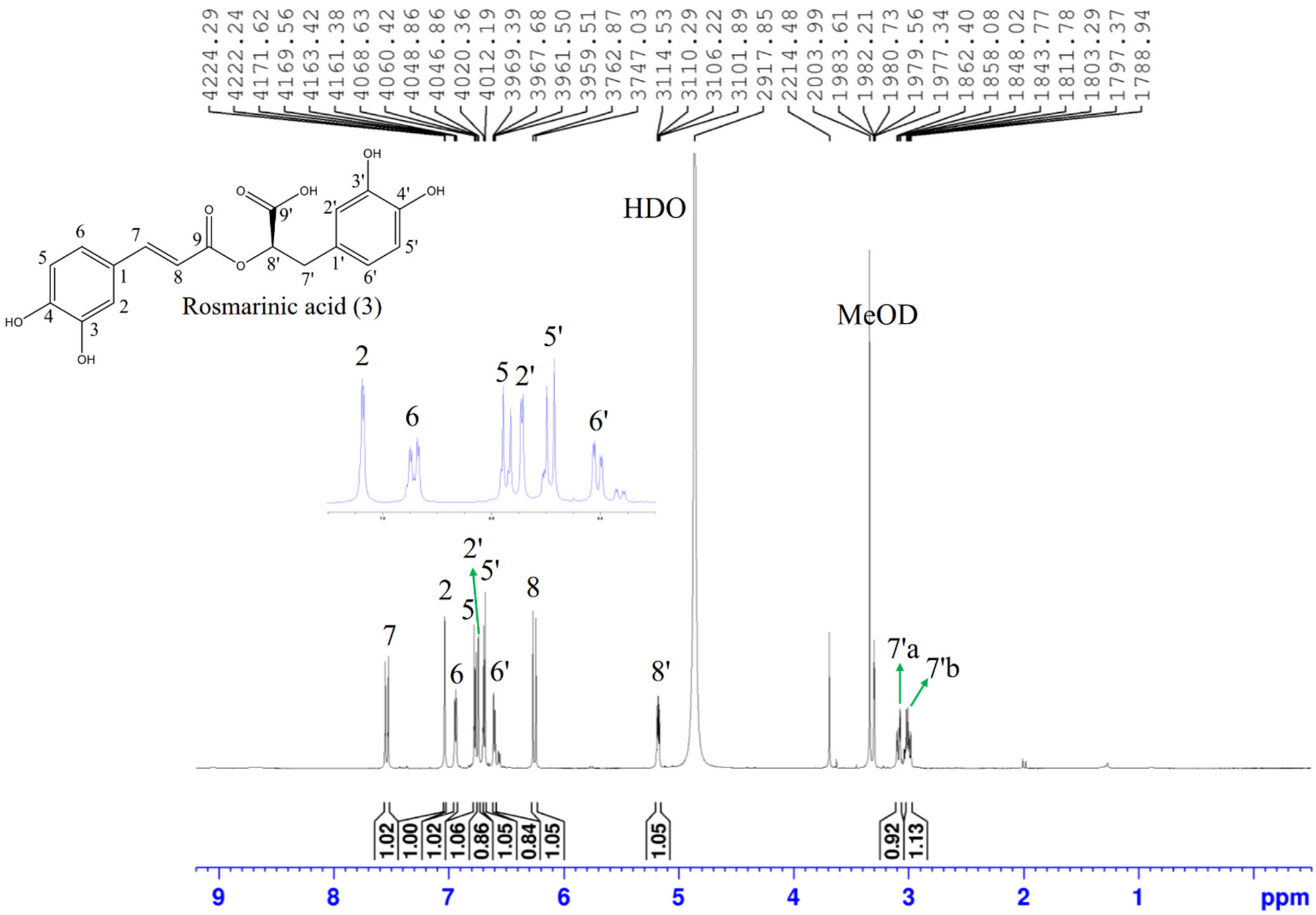

Figure S5.1. ^{1}H-NMR (600 MHz, MeOH-d_4) spectroscopy of rosmarinic acid (**3**).

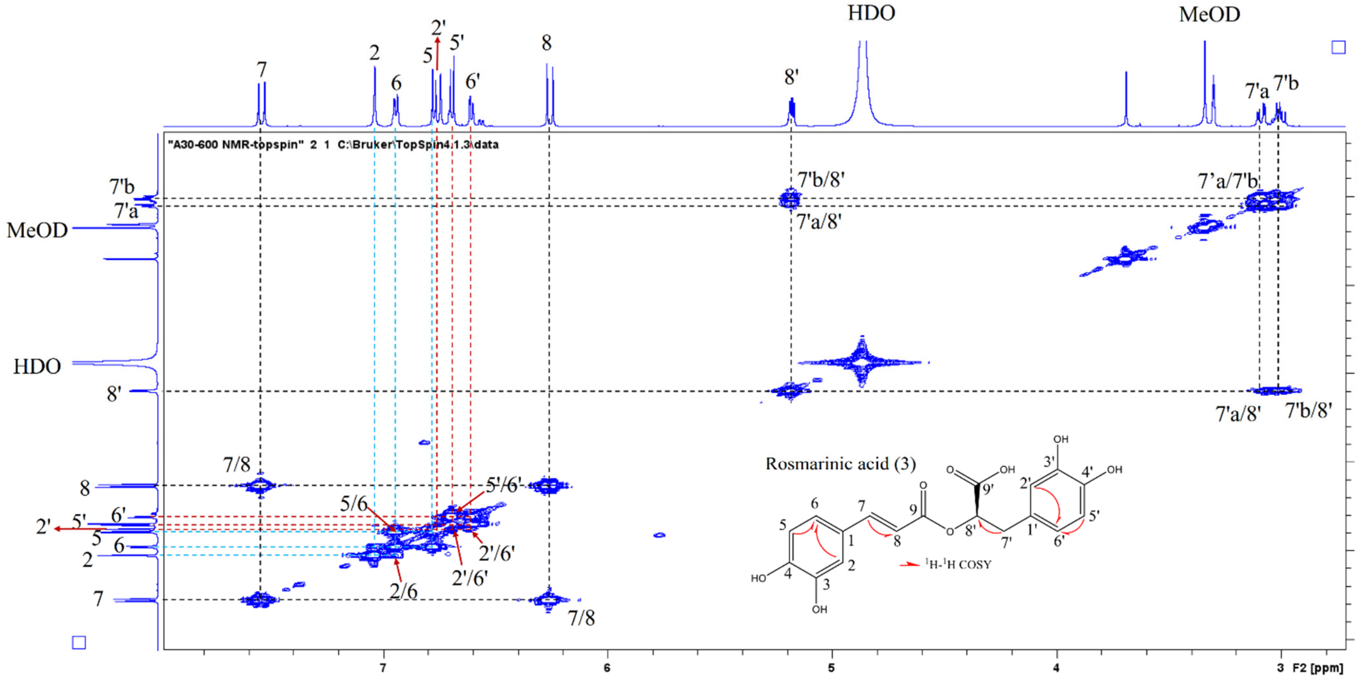

Figure S5.2. ^{1}H-^{1}H COSY NMR (600 MHz, MeOH-d_4) spectroscopy of rosmarinic acid (**3**).

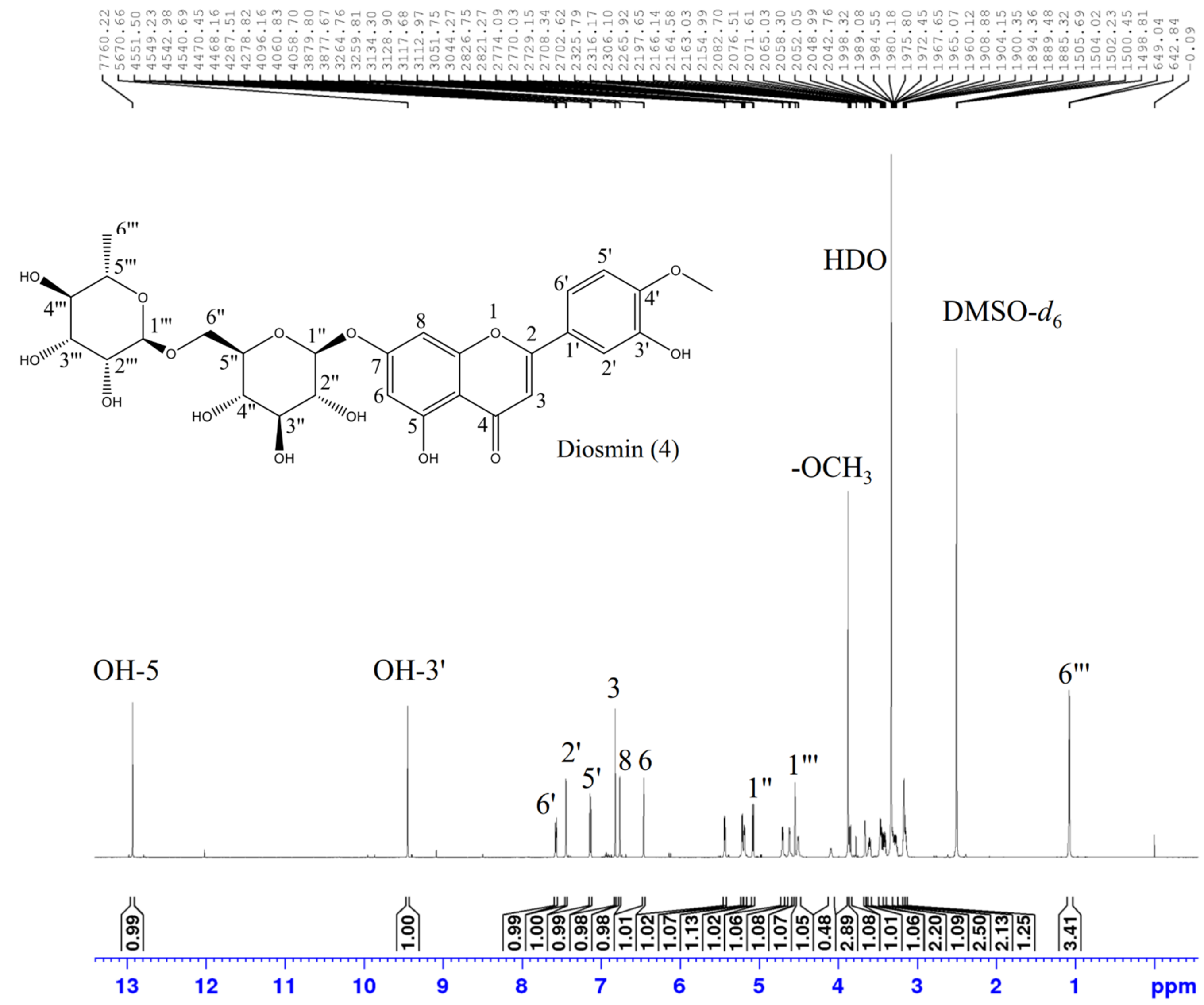

Figure S6.1. Part 1 of ^{1}H-NMR (600 MHz, DMSO-d_6) spectroscopy of diosmin (**4**).

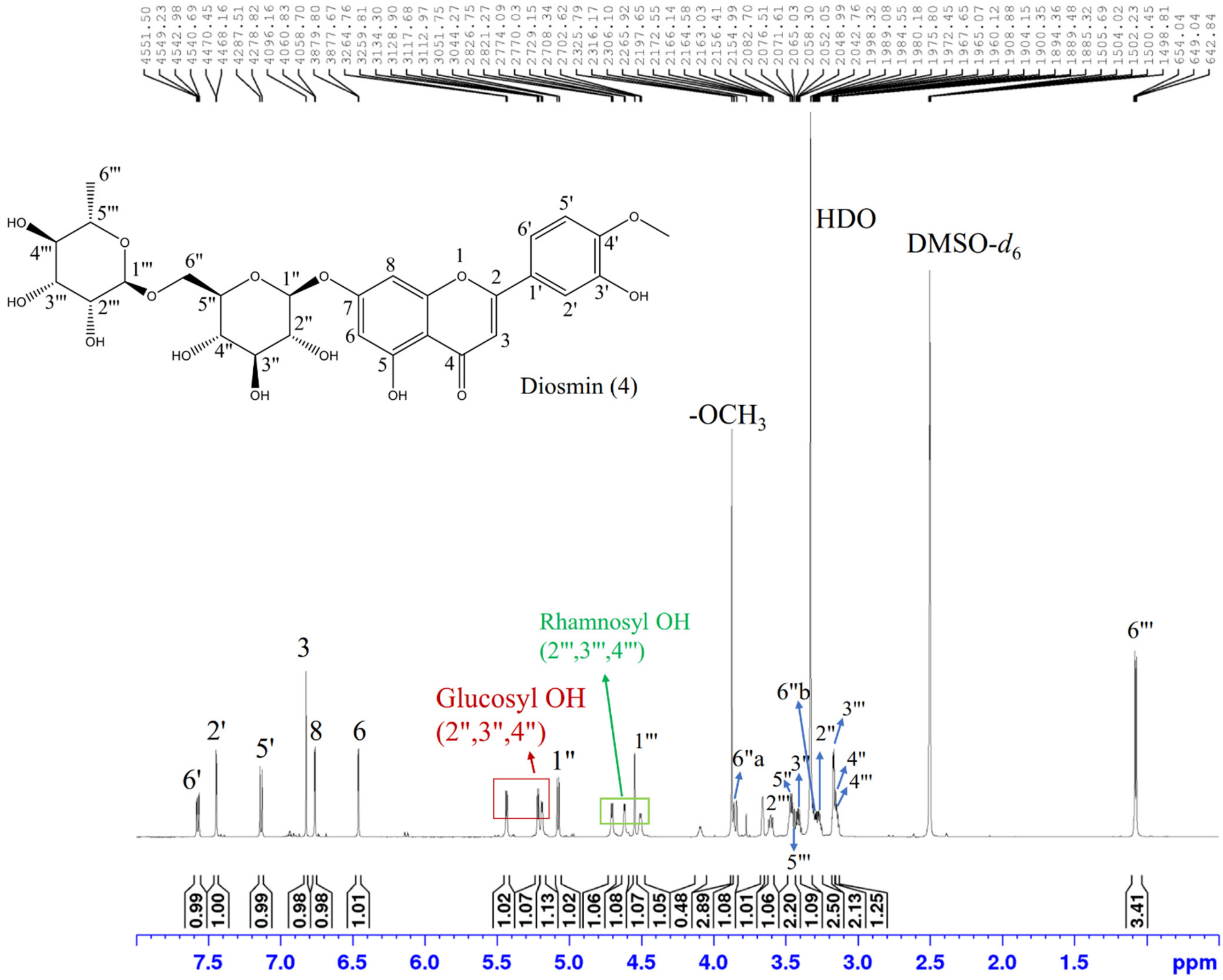

Figure S6.2. Part 2 of ^{1}H-NMR (600 MHz, DMSO-d_6) spectroscopy of diosmin (**4**). Please note that the assignment labels of the severely overlapped sugar protons between 3.0 ppm and 3.5 ppm for compound **4** were tentatively assigned according to its ^{1}H-^{1}H COSY NMR data.

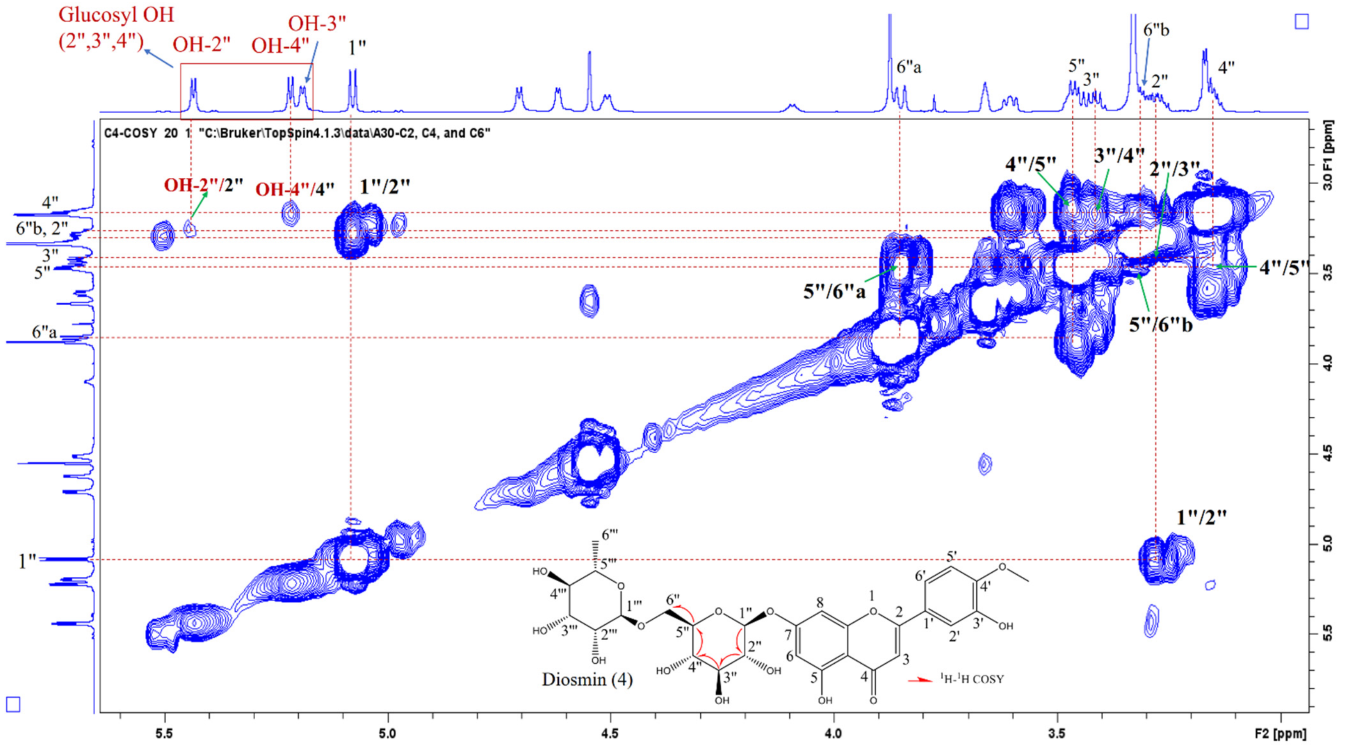

Figure S6.3. Part 1 of ^{1}H-^{1}H COSY NMR (600 MHz, DMSO-d_6) spectroscopy of diosmin (**4**). Please note that the assignment labels of the severely overlapped sugar protons between 3.0 ppm and 3.5 ppm for compound **4** were tentatively assigned according to the crosspeaks in its ^{1}H-^{1}H COSY NMR data.

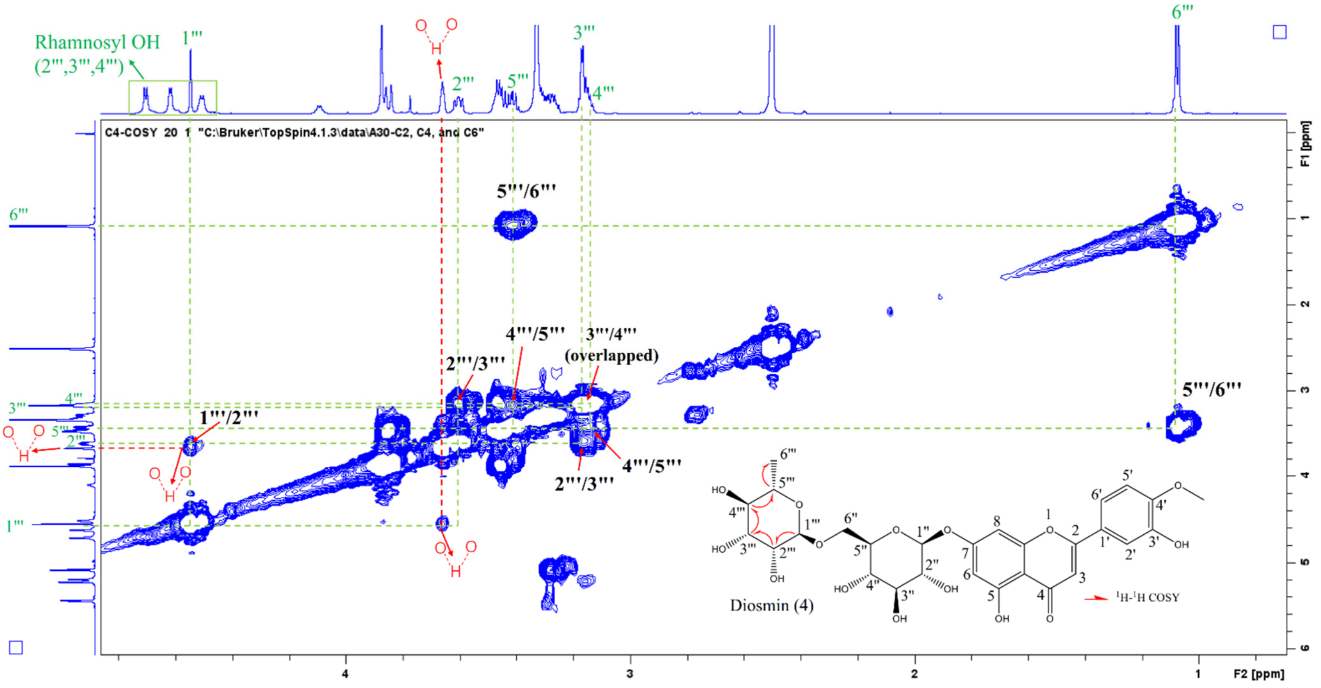

Figure S6.4. Part 2 of ^{1}H-^{1}H COSY NMR (600 MHz, DMSO-d_6) spectroscopy of diosmin (**4**). Please note that the assignment labels of the severely overlapped sugar protons between 3.0 ppm and 3.5 ppm for compound **4** were tentatively assigned according to the crosspeaks in its ^{1}H-^{1}H COSY NMR data.

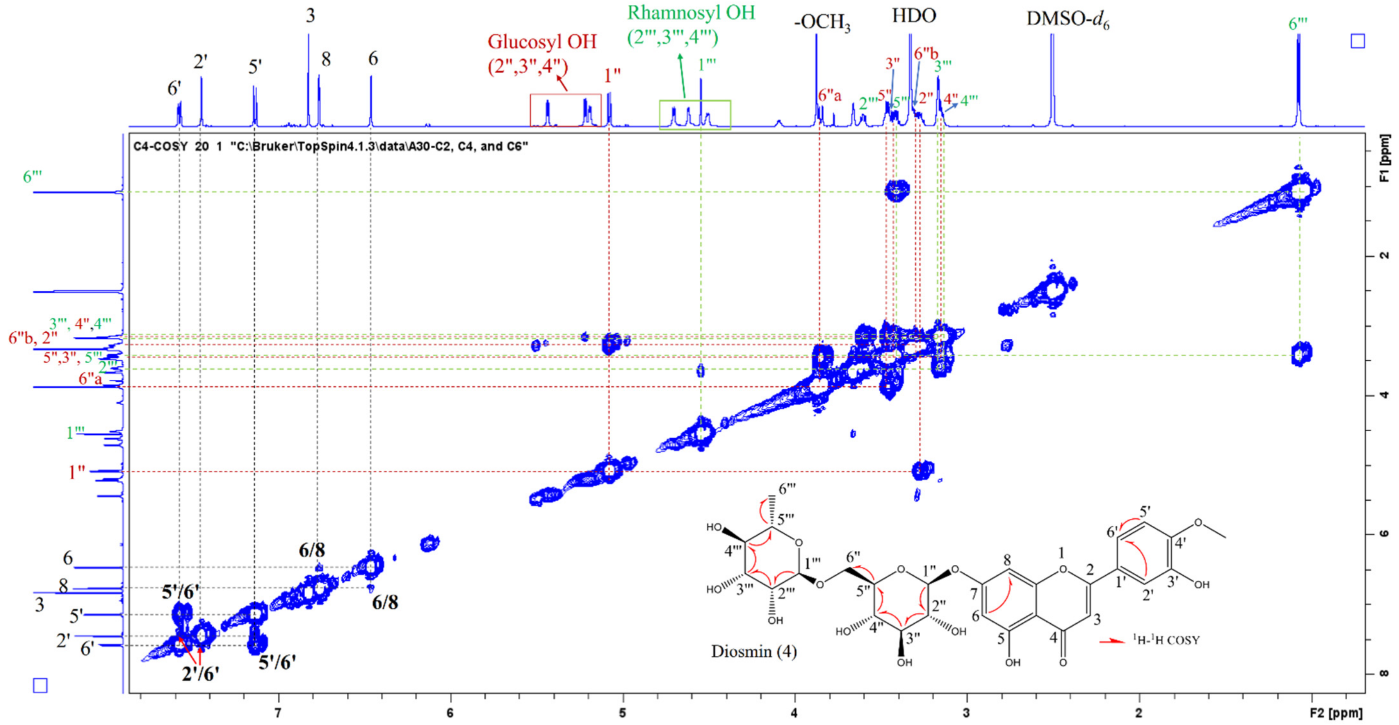

Figure S6.5. Part 3 of ^{1}H-^{1}H COSY NMR (600 MHz, DMSO-*d*6) spectroscopy of diosmin (**4**). Please note that the assignment labels of the severely overlapped sugar protons between 3.0 ppm and 3.5 ppm for compound **4** were tentatively assigned according to the crosspeaks in its ^{1}H-^{1}H COSY NMR data.

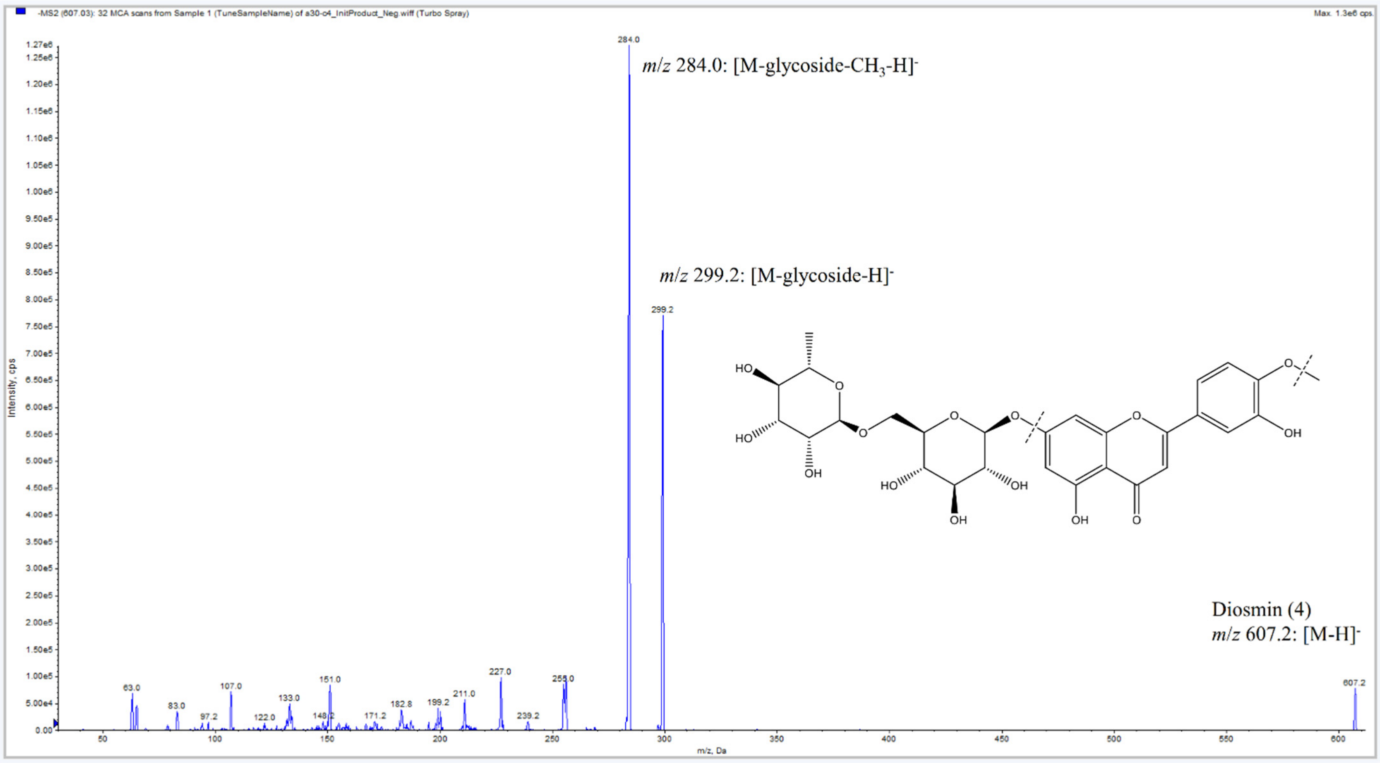

Figure S6.6. ESI-MS/MS (negative ion) spectroscopy of diosmin (**4**).

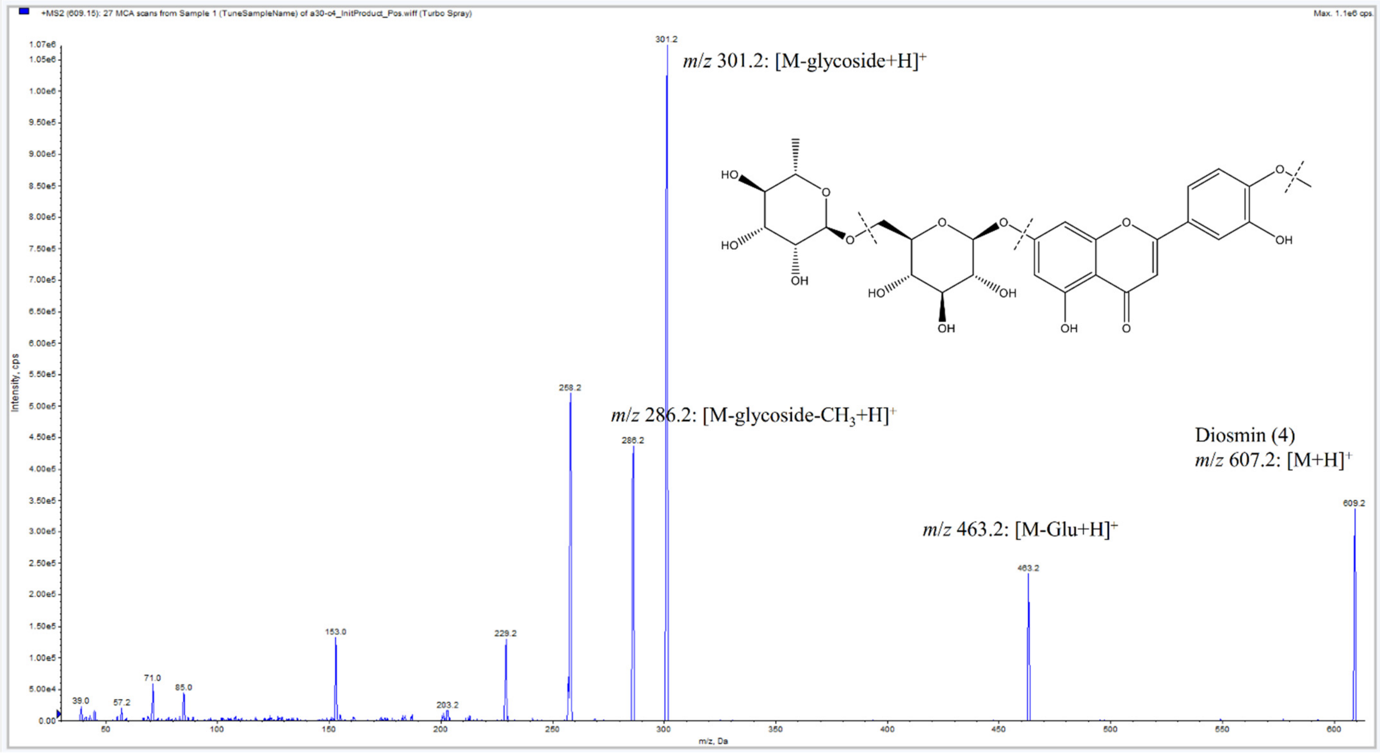

Figure S6.7. ESI-MS/MS (positive ion) spectroscopy of diosmin (**4**).

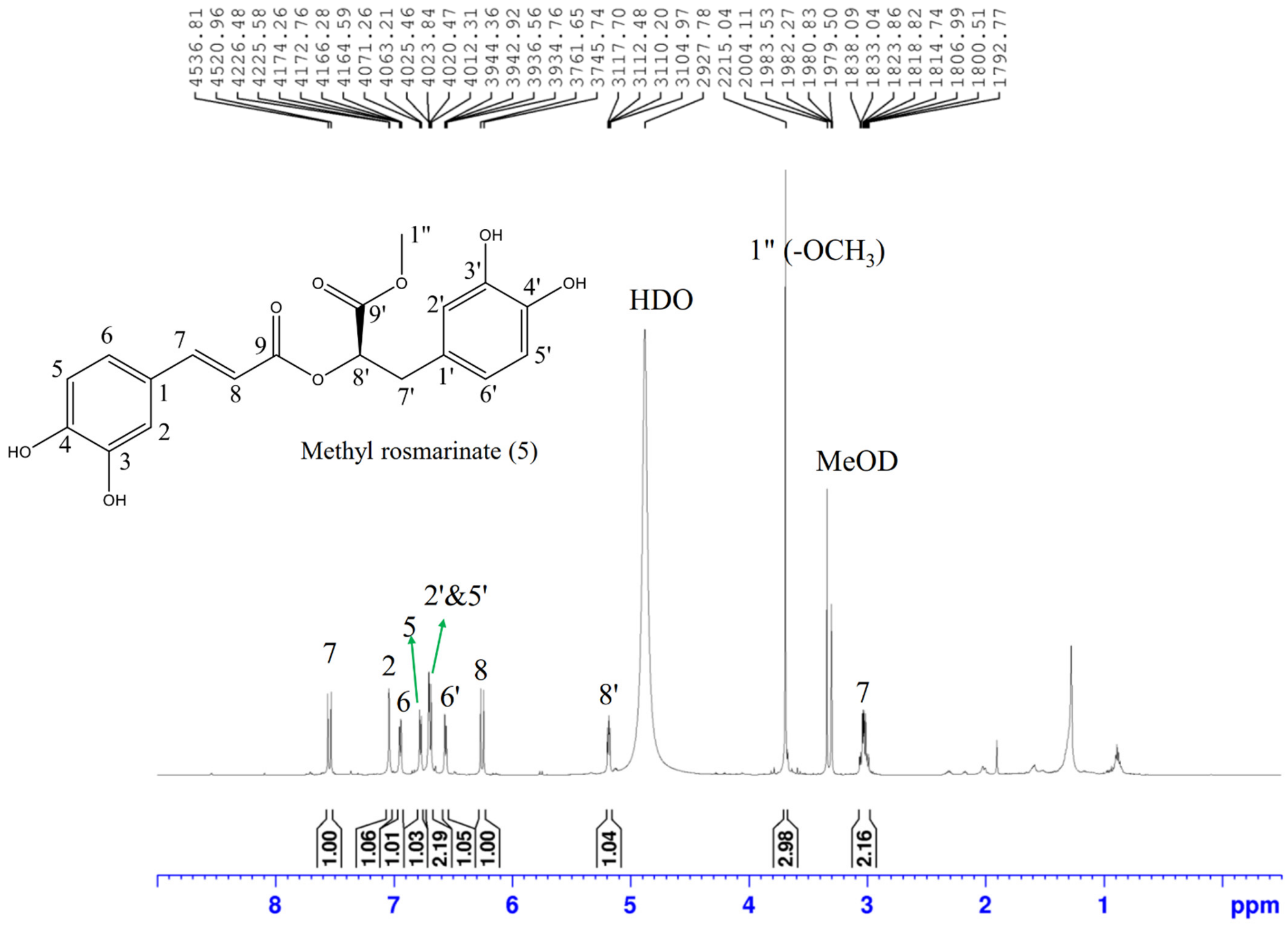

Figure S7. ^{1}H-NMR (600 MHz, MeOH-d_4) spectroscopy of methyl rosmarinate (**5**).

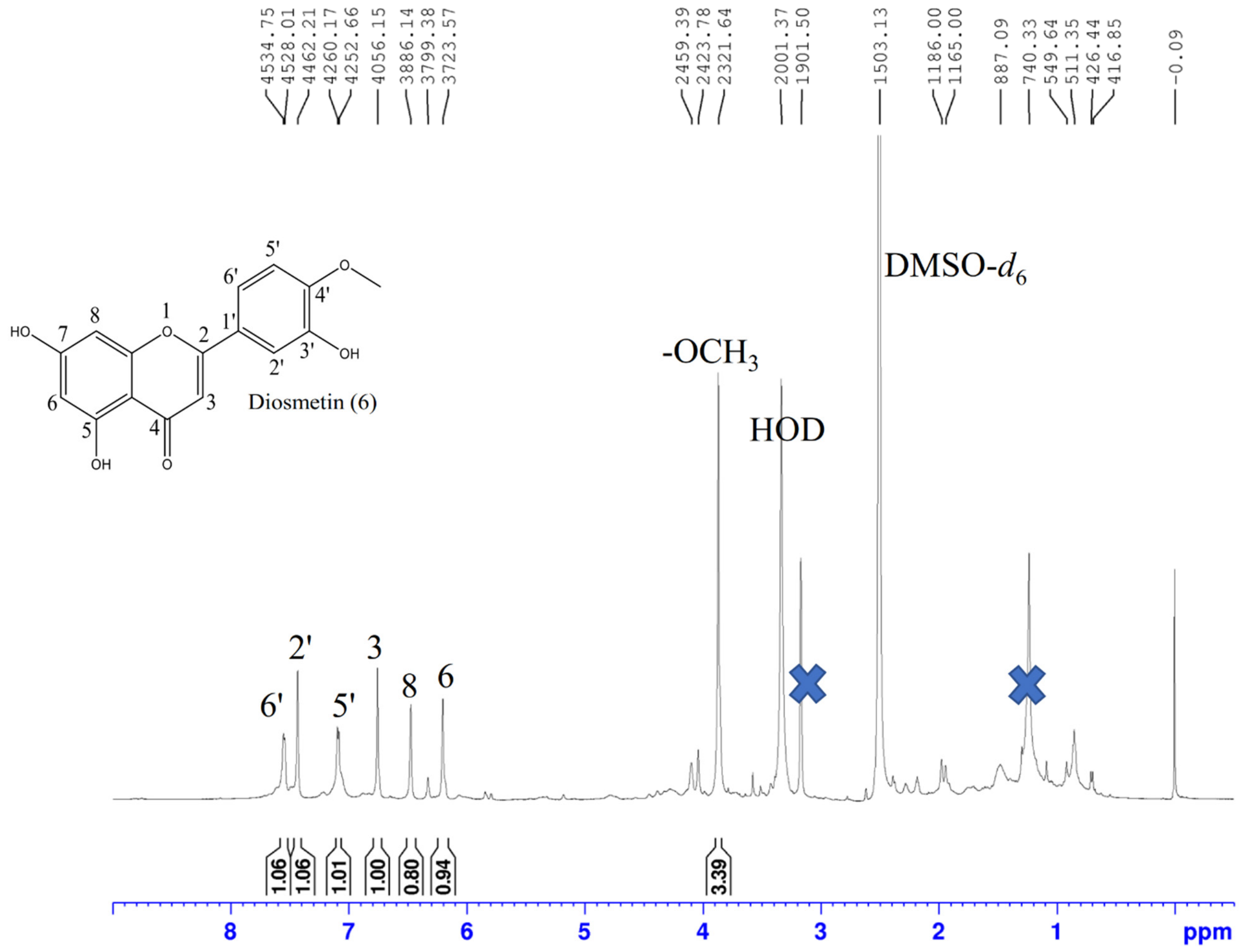

Figure S8.1. ^{1}H-NMR (600 MHz, DMSO-d_6) spectroscopy of diosmetin (**6**).

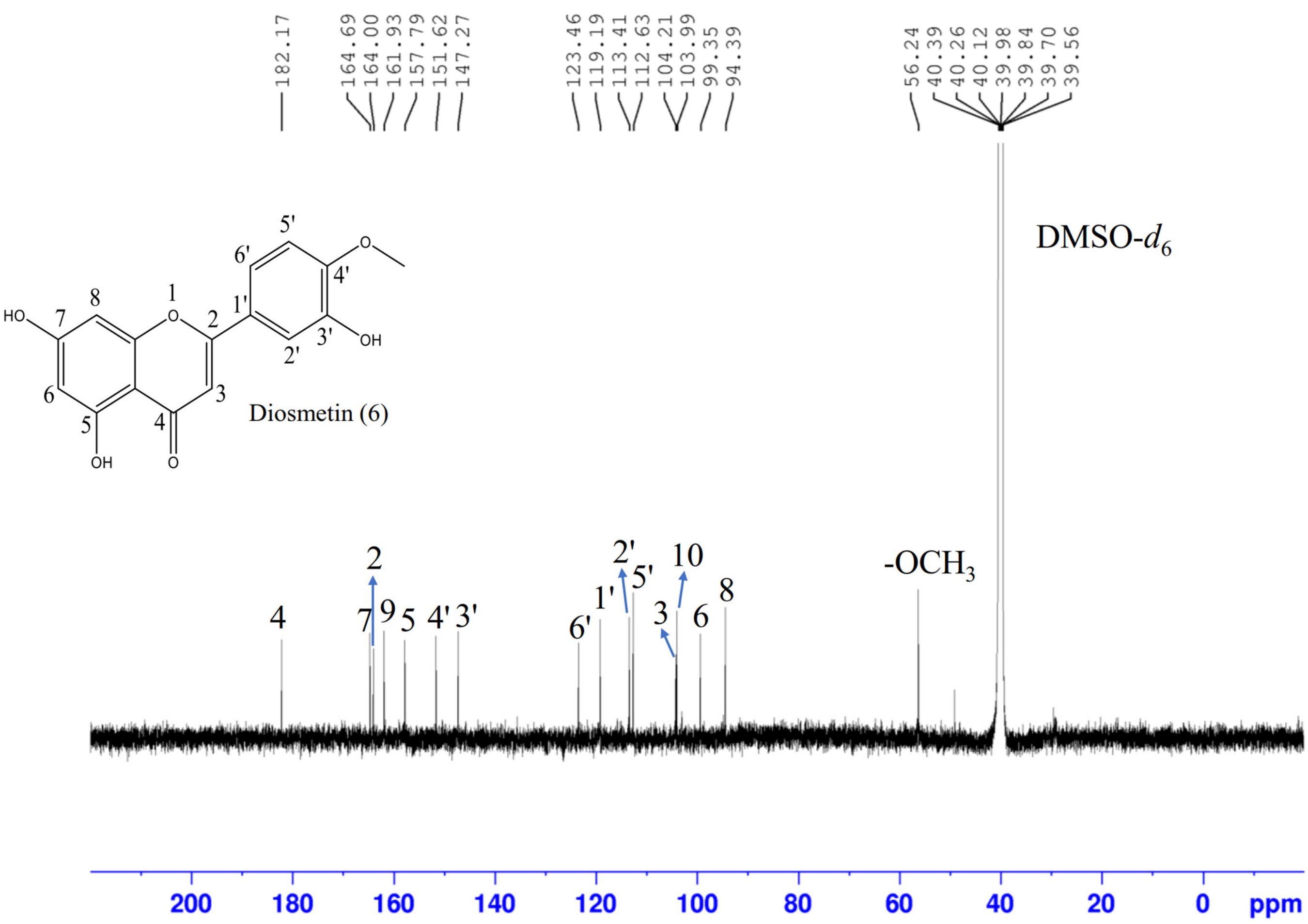

Figure S8.2. ^{13}C-NMR (150 MHz, DMSO-d_6) spectroscopy of diosmetin (**6**).

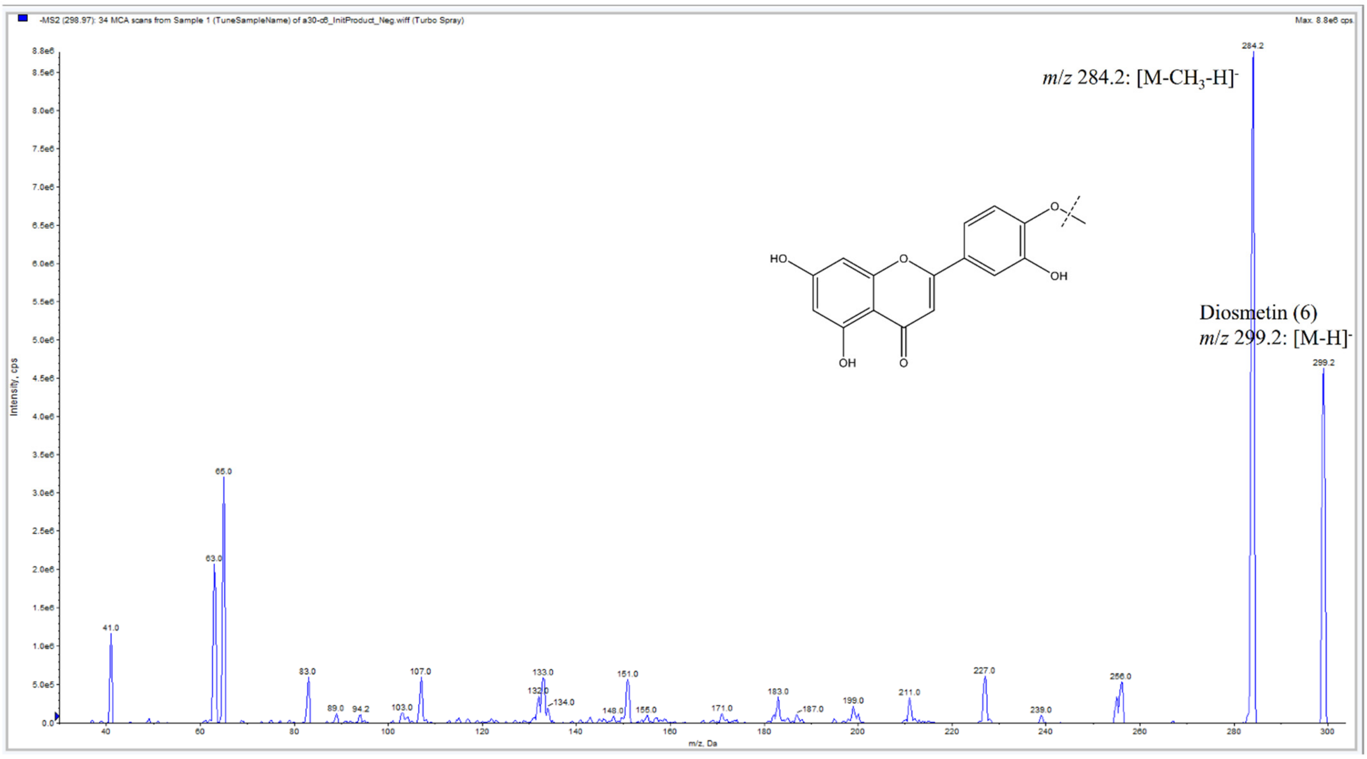

Figure S8.3. ESI-MS/MS (negative ion) spectroscopy of diosmetin (**6**).

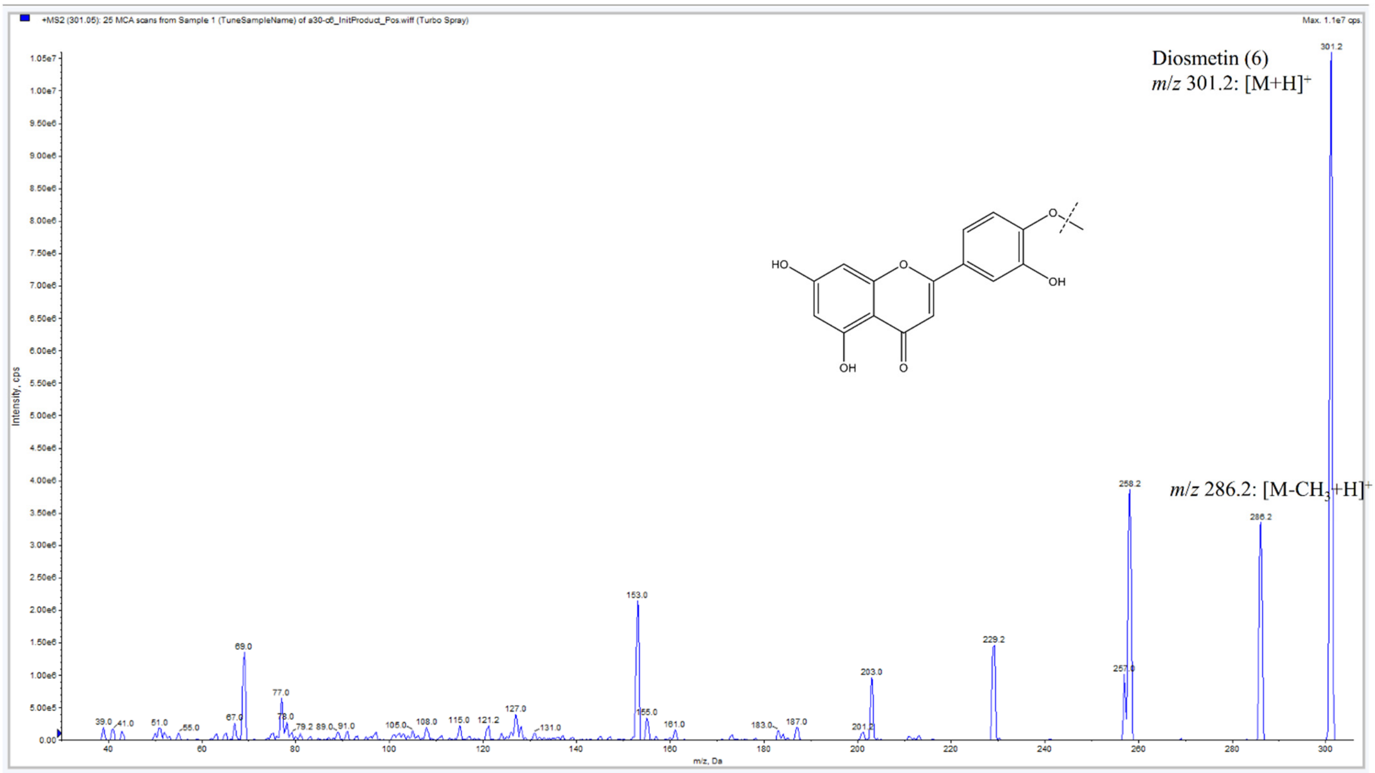

Figure S8.4. ESI-MS/MS (positive ion) spectroscopy of diosmetin (**6**).

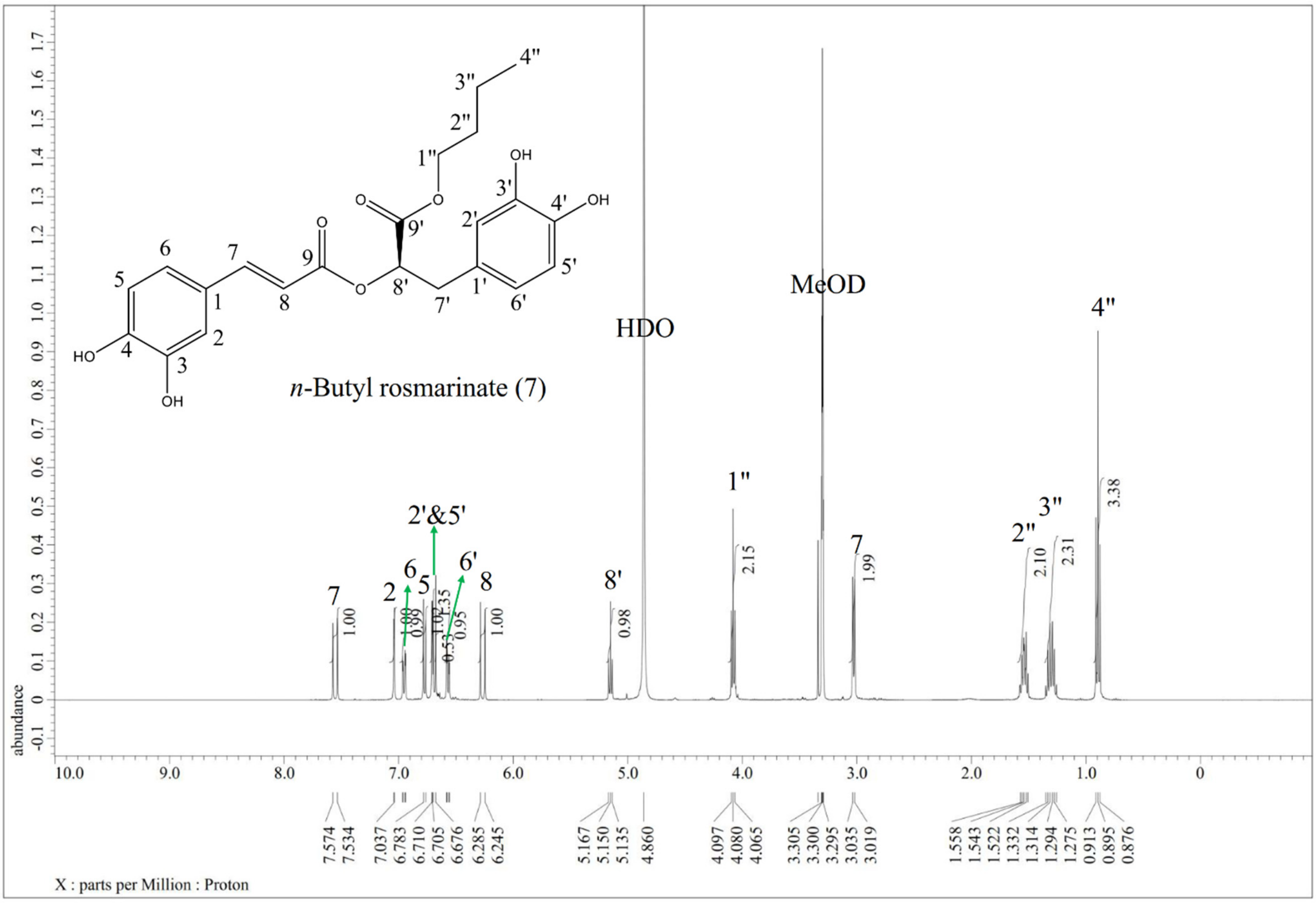

Figure S9. ^{1}H-NMR (400 MHz, MeOH-d_4) spectroscopy of *n*-butyl rosmarinate (**7**).

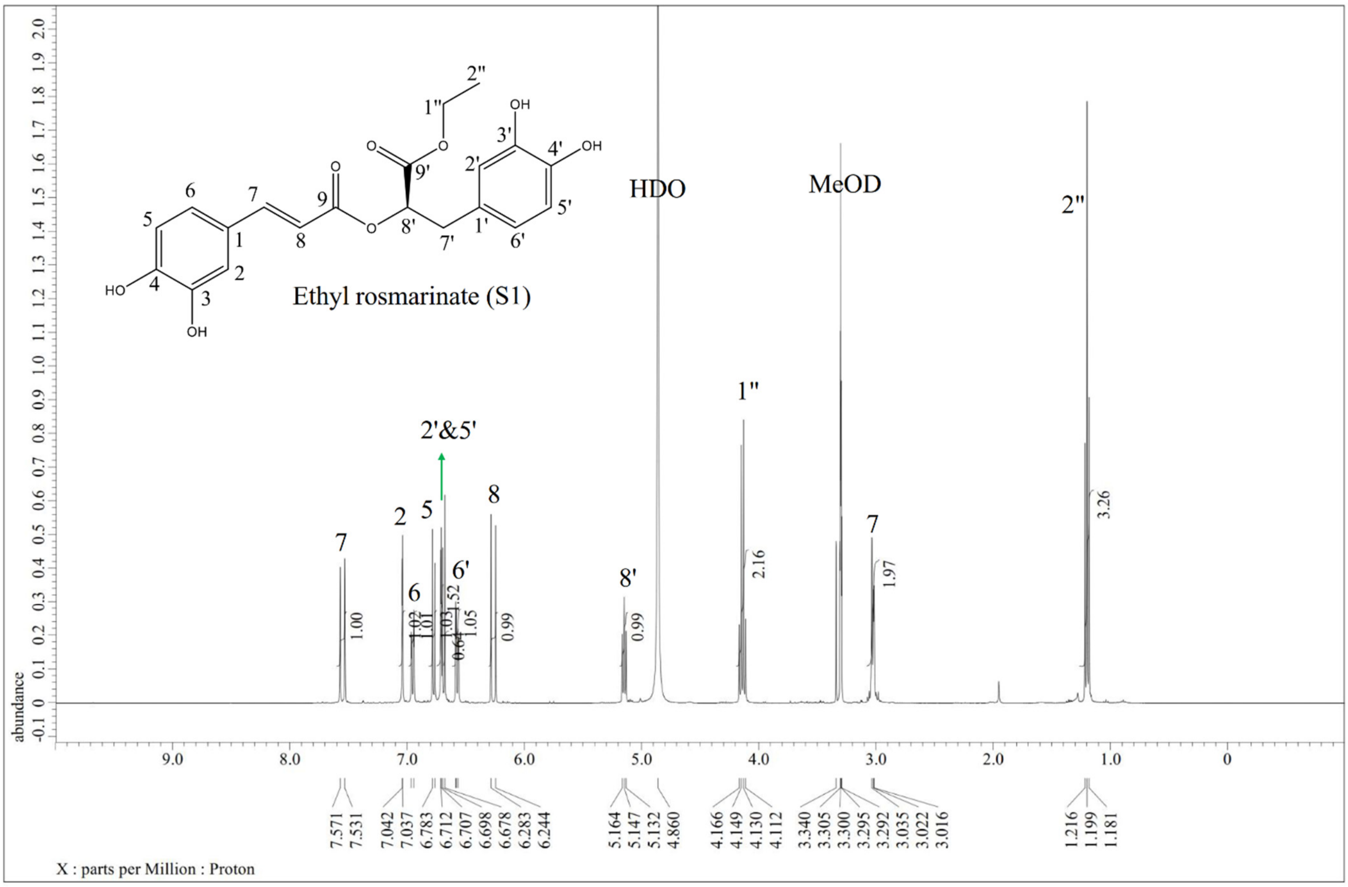

Figure S10. ^{1}H-NMR (400 MHz, MeOH-d_4) spectroscopy of synthesized ethyl rosmarinate (**S1**).

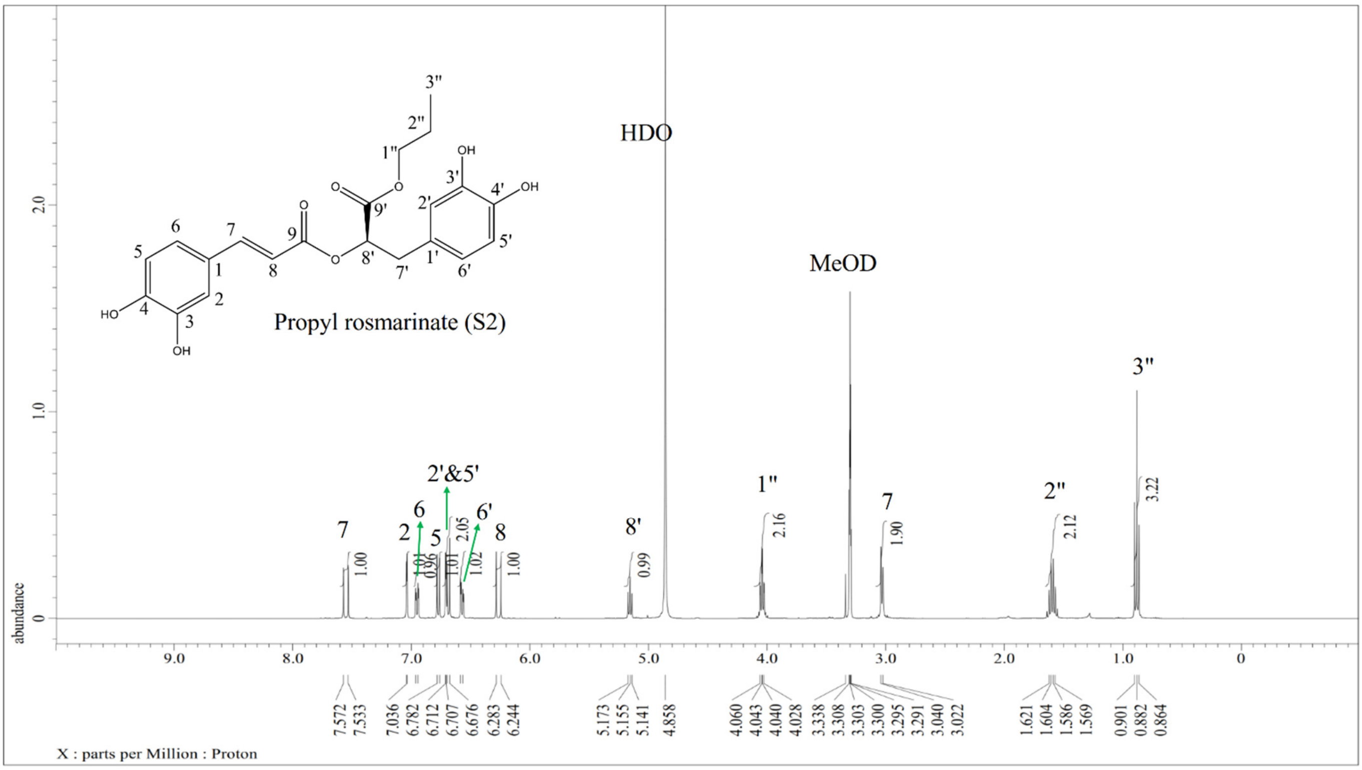

Figure S11. ^{1}H-NMR (400 MHz, MeOH-d_4) spectroscopy of synthesized propyl rosmarinate (**S2**).
